# Aged gut microbiota promotes arrhythmia susceptibility via oxidative stress

**DOI:** 10.1016/j.isci.2024.110888

**Published:** 2024-09-04

**Authors:** Zhi-ping Fu, Yi-ge Ying, Rui-yao Wang, Yu-qing Wang

**Affiliations:** 1 Collage of Pharmacology, North China University of Science and Technology, Tangshan 063200, China

**Keywords:** Cardiovascular medicine, Microbiome

## Abstract

Arrhythmias and sudden cardiac death (SCD) impose a significant burden. Their prevalence rises with age and is linked to gut dysbiosis. Our study aimed to determine whether aged gut microbiota affects arrhythmogenesis. Here, we demonstrated that arrhythmia susceptibility in aged mice could be transmitted to young mice using fecal microbiota transplantation (FMT). Mechanistically, increased intestinal reactive oxygen species (ROS) in aged mice reduced ion channel protein expression and promoted arrhythmias. Gut microbiota depletion by an antibiotic cocktail reduced ROS and arrhythmia in aged mice. Interestingly, oxidative stress in heart induced by hydrogen peroxide (H_2_O_2_) increased arrhythmia. Moreover, aged gut microbiota could induce oxidative stress in young mice colon by gut microbiota metabolites transplantation. Vitexin could reduce aging and arrhythmia through OLA1-Nrf2 signaling pathway. Overall, our study demonstrated that the gut microbiota of aged mice reduced cardiac ion channel protein expression through systemic oxidative stress, thereby increased the risk of arrhythmias.

## Highlights

• The dysbiosis associated with aging increases levels of reactive oxygen species
• The dysbiosis promotes age-related arrhythmogenesis
• Oxidative stress reduced the expression of ion channel proteins in heart
• Vitexin decrease aging-related arrhythmia through OLA1-Nrf2 signaling pathway

## Graphical abstract

**Figure:**
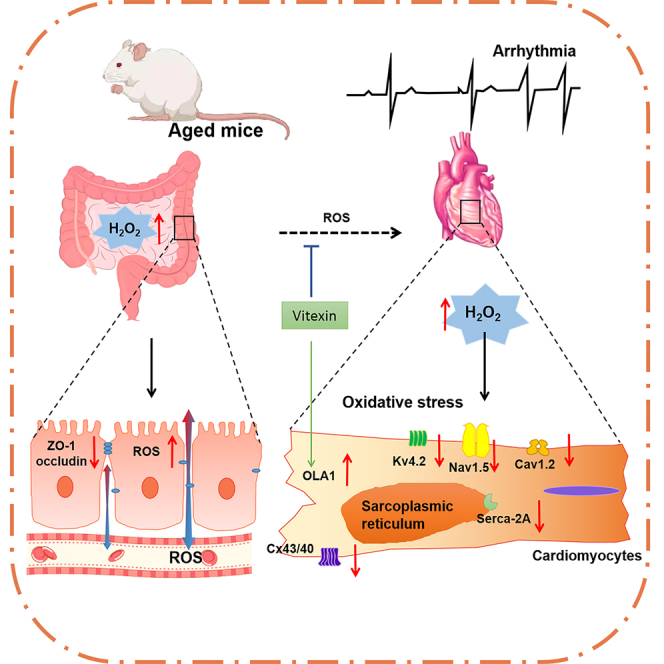

## Introduction

About 18 million people die from cardiovascular diseases (CVD) worldwide every year, with half of them experiencing sudden cardiac death (SCD). The primary cause of SCD is ventricular arrhythmia caused by CVD. Arrhythmia is an abnormal electrical activity of the heart that leads to too fast, too slow, or irregular heartbeat, which can affect the heart’s diastole and systole function and cause many health problems. Cardiac arrhythmias are mainly caused by multiple risk factors, such as organic heart disease, dysregulation of the nervous and endocrine systems, and electrolyte imbalance, which significantly contribute to morbidity and mortality.^1^^,^^2^ Advanced age is one of the important independent risk factors of arrhythmia. The prevalence of arrhythmias and incidence of SCD increases markedly with age, leading to higher morbidity and mortality in the elderly.^3^ The frequency of arrhythmias, particularly atrial fibrillation (AF) and ventricular tachyarrhythmias (VT), is expected to increase with aging. Additionally, the occurrence of aging-related arrhythmia remains high due to complex molecular mechanisms and severe complications.^4^ Therefore, it is urgent to understand the pathogenesis of aging-related arrhythmia and find therapeutic strategies to promote its early prevention, diagnosis, and treatment.

Gut microbiota has been recognized as an important factor in the development of cardiovascular diseases, such as AF, heart failure (HF), ischemia, and reperfusion. It is also considered to be an endocrine organ that plays a role in the manipulation of host immunity and metabolic homeostasis.^5^ It has been shown to play both beneficial and adverse roles in AF rats and patients, which could be related to the heterogeneity of the microbiota.^6^ Researches indicate that in patients with AF, the relative abundance of Ruminococcus, Streptococcus, and Enterococcus increase, while that of Faecalibacterium, Oscillospira, and Bilophila decreases. From the perspective of intestinal content metabolism, the bacteria producing trimethylamine oxide (TMAO) in gut microbiota of AF patients increase. Moreover, local injection of TMAO in canine can activate atrial autonomic nerve plexus, shorten ERP value, and then promote arrhythmia, which because of the activation of p65/NF-κB signaling and inflammatory cytokines.^7^ Studies showed that specific intestinal bacteria and bioactive metabolites (such as ruminococcus, goose deoxycholic acid, Prevotella copri, Prevotella copri CAG:164, and α-linolenic acid) was found in patients with paroxysmal AF and persistent AF, and they are considered to be directly involved in the progression of AF or at least biomarkers of AF. And, there is a correlation between choline, a lipid phosphatidylcholine metabolite of intestinal flora, and atrial diameter.^8^ Accumulating evidences indicate that the composition and regulation of gut flora in the elderly is different from that in youth, which is referred to as gut dysbiosis in elderly.^2^^,^^3^^,^^4^ A previous study identified variable metabolites as well as an imbalanced gut microbiota composition in patients with AF.^9^ Aging-related gut dysbiosis accelerates aging and leads to oxidative stress in the host.^10^^,^^11^^,^^12^ However, the exact mechanism underlying aging-related microbial dysbiosis and oxidative stress contribute to arrhythmias during aging remains unclear, requiring more experimental evidences.^10^^,^^11^^,^^12^^,^^13^ Many gut microbiota metabolites are directly associated with increased ROS, which is one of the most important risk factors for arrhythmias.^11^^,^^14^ Numerous experimental and clinical studies suggested that oxidative stress and inflammation were detrimental to cardiovascular health and could increase heart susceptibility to arrhythmias. Increased oxidative stress was also a crucial factor in cellular senescence and aging.^15^ Additionally, oxidative stress adversely affects ion homeostasis by altering the structure and electrical function of cardiac ion channels. Therefore, we hypothesized that ROS from colon might have implications for increased susceptibility to age-related arrhythmias, which might due to alterations in ROS mediated by dysbiosis in aged mice.

In this study, we explored the causal relationship between the aging gut microbiota and aging-related arrhythmia. We have demonstrated that metabolites produced by aged gut flora regulated arrhythmogenesis by ROS derived from colon. It helped to regulate oxidative stress in the heart, which in turn promotes reduced ion channel protein expression. Next, we explored the impacts of aged gut microbiota depletion on cardiac electrophysiology, and we found it attenuated susceptibility to arrhythmia by reducing oxidative stress. Our results showed that gut dysbiosis in aged mice increased ROS, which in turn promoted aging-related arrhythmia by reducing cardiac ion channel proteins expression. Through fecal microbiota metabolites transplantation (FMMT), we found a previously undescribed role for colonic ROS in promoting decreased ion channel protein expression through oxidative stress. Furthermore, we observed that vitexin reduced the susceptibility to aging-related arrhythmias induced by ROS, and improve cardiac ion channel expression. These findings highlight the role of discovered gut microbiota metabolites in regulating cardiac oxidative stress and the expression of cardiac ion channel proteins, thereby increasing susceptibility to arrhythmias. This study provides pathophysiology insights into the causal relationship between gut microbiota and arrhythmias in the elderly and opens up avenues for the treatment of aging-related arrhythmias.

## Result

### Aged microbial transplantation increased arrhythmia susceptibility in young mice

**Figure 1:**
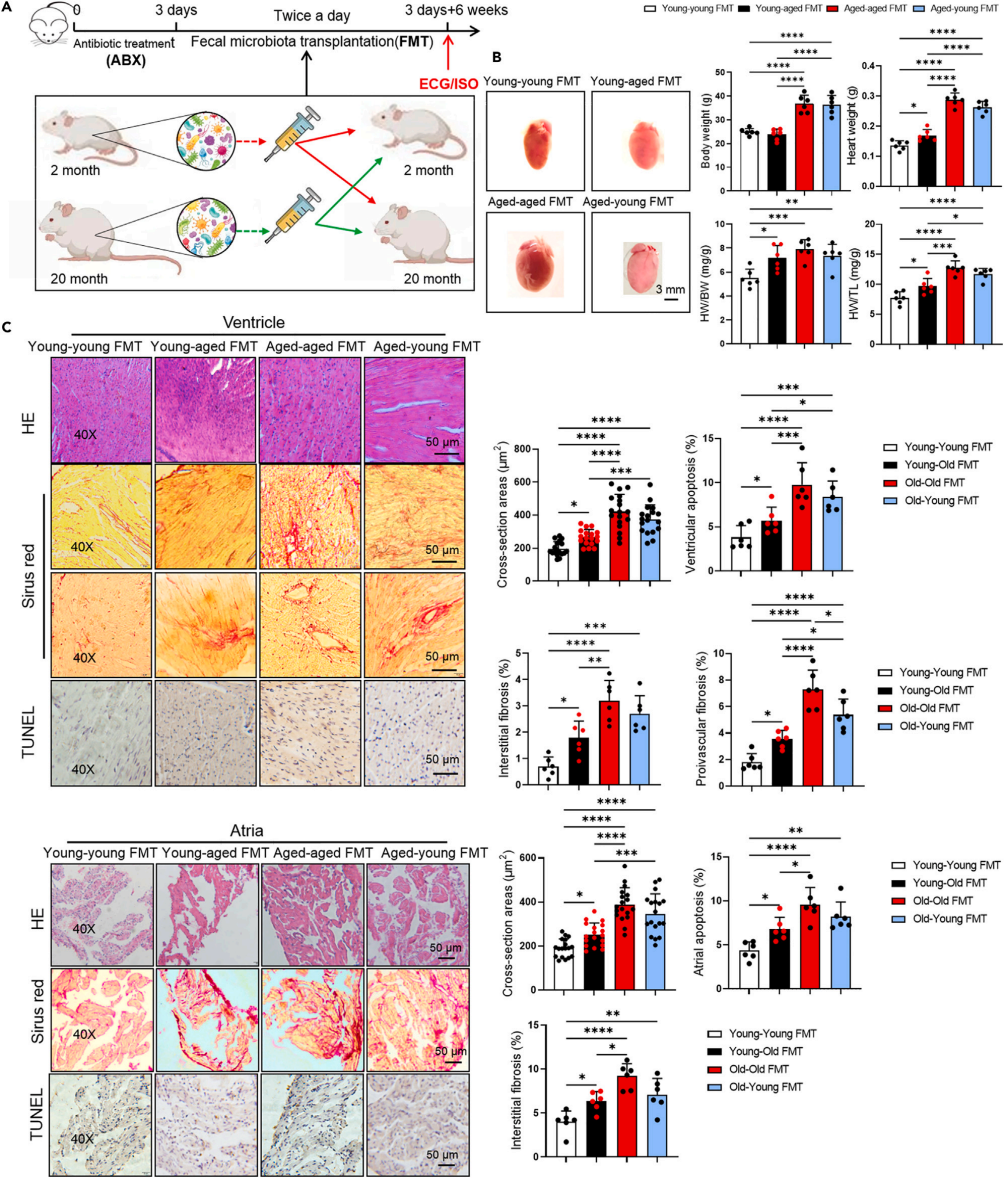
Effect of gut microbiota transplantation on cardiac structure (A) Experimental design for testing the effect of gut microbiota transplantation on young and aged mice. (B) The overall heart size. Scale bar, 3 mm. Quantitative analysis of body weight (2-month and 20-month old), heart weight, heart/body weight (HW/BW), heart weight/tibia length (HW/TL) in the different groups (*n* = 6/per group). Data are presented as the mean ± SD. Data analyzed by one-way ANOVA with Tukey’s post-hoc test. ∗*p* < 0.05, ∗∗*p* < 0.01, ∗∗∗*p* < 0.001, ∗∗∗∗*p* < 0.0001. (C) Representative H&E, Sirius red, TUNEL staining images and interstitial and perivascular fibrosis in the ventricular and atria (*n* = 6/per group). Scale bar, 50 μm. Young fecal microbiota transplantation attenuated atrial fibrosis in aged mice measured by Sirius red staining. Data are presented as the mean ± SD. Data analyzed by one-way ANOVA with Tukey’s post-hoc test. ∗*p* < 0.05, ∗∗*p* < 0.01, ∗∗∗*p* < 0.001, ∗∗∗∗*p* < 0.0001. FMT indicates fecal microbiota transplantation; young, 2-months old mice; old, old 20-months old mice; young-old FMT, transplantation of gut microbiota from old mice to young mice.

**Figure 2:**
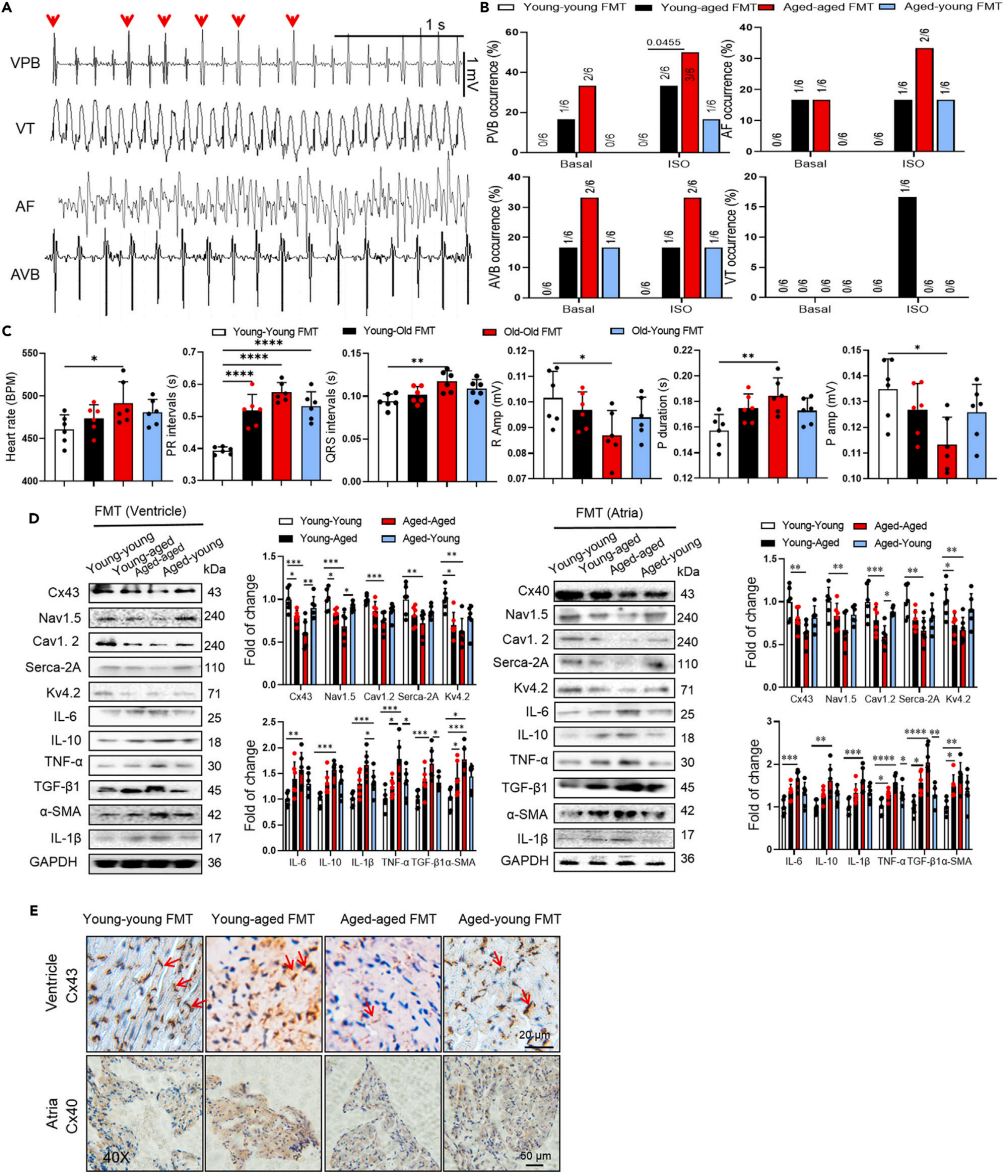
Transplantation of gut microbiota from aged mice increases susceptibility to arrhythmia (A) Sample ECG traces of four different types of arrhythmic events: ventricular premature beats (VPB), ventricular tachycardia (VT), atrial fibrillation (AF), and atrioventricular block (AVB). (B) Summary the inducibility of VPB, VT, AF, and AVB in young and aged mice. Numbers in parentheses indicate the number of mice that were induced into VPB, VT, AF, AVB following ISO treated (*n* = 6/per group). Data analyzed by Fischer’s exact test. (C) Differences in heart rates, PR intervals, QRS intervals, R amplitude, P duration and P amplitude among young-young FMT, young-aged FMT, aged-aged FMT and aged-young FMT mice (*n* = 6/per group). Data are presented as the mean ± SD. Data analyzed by one-way ANOVA with Tukey’s post-hoc test. ∗*p* < 0.05, ∗∗*p* < 0.01, ∗∗∗*p* < 0.001, ∗∗∗∗*p* < 0.0001. (D) Representative western blot and quantification of Cx43, Cx40, Nav1.5, Cav1.2, Serca-2A, Kv4.2, IL-6, IL-10, IL-1β, TNF-α, TGF-β, α-SMA, GAPDH in ventricular and atrial tissue of young-young FMT, young-aged FMT, aged-aged FMT and aged-young FMT mice (*n* = 6/per group). GAPDH was used for internal normalization. Data are presented as the mean ± SD. Data analyzed by one-way ANOVA with Tukey’s post-hoc test. ∗*p* < 0.05, ∗∗*p* < 0.01, ∗∗∗*p* < 0.001, ∗∗∗∗*p* < 0.0001. (E) Representative micrographs of Cx43 signal in ventricular and Cx40 in stained atrial sections visualized (*n* = 6/per group). Cx43 indicates Connexin 43; Cx40, Connexin 40; ISO, isoproterenol; IL-6, Interleukin-6; TNF-α, tumor necrosis factor-α; TGF-β, transforming growth factor β.

Susceptibility to arrhythmia increases with age.^16^ Similarly, gut microbiota undergoes changes with age, but it remains unclear to what extent these changes are attributed to age-related inflammation, medications, or chronic diseases.^17^ To investigate the role of gut microbiota in the age-related arrhythmogenesis, we performed fecal microbiota transplantation (FMT) to young (2-month old) and aged mice (20-month old) for 6 weeks (Figure 1A). We transplanted the gut microbiota of old mice into themselves (aged-aged FMT) as well as into young mice (aged-young FMT), and similarly, we transplanted the gut microbiota of young mice into themselves (young-young FMT) as well as into old mice (young-aged FMT). There was no difference in body weight among the 4 groups (Figure 1A). But, we found that young-aged FMT mice showed a reduction in body weight. Heart weight (*p* = 0.0337), heart weight-to-tibia length (HW/TL, *p* = 0.0318), and heart weight-to-body weight (HW/BW, *p* = 0.0194) ratio were increased in young-aged FMT mice compared with young mice (Figures 1A and 1B). Consistent with this, analyses of H&E, Sirius red, and TUNEL staining of histological sections showed increased interstitial (*p* = 0.0345 in ventricle, *p* = 0.0449 in atria) and perivascular fibrosis (*p* = 0.0497 in ventricle), as well as apoptosis (*p* = 0.0359 in ventricle, *p* = 0.0494 in atria) in young-aged FMT mice when compared with young mice (Figure 1C). A decreased perivascular fibrosis (*p* = 0.028) was found in aged-young FMT mice compared to aged mice. Measurements of myocyte cross-sectional area revealed that myocytes of young-aged FMT mice were significantly larger in ventricle (*p* = 0.0422) and atria (*p* = 0.0466) when compared with young mice (Figure 1C). The previous results indicated that the gut microbiota from young mice protected from cardiac aging, while the gut microbiota of aged mice promoted it. As expected, we found arrhythmia after administrated 2 mg/kg isoproterenol (ISO, i.p.) in the aged-aged FMT, aged-young FMT, and young-aged FMT mice, but not in young mice (Figure 2A). The incidence of premature ventricular beats (PVB), AF and atrioventricular block (AVB) in aged mice was higher than other mice. Interestingly, VT was only exhibited in young-aged FMT mice (Figure 2B). The occurrence of arrhythmia and ISO-induced arrhythmia were reduced in aged-young FMT mice (Figure 2B). And aged gut microbiota also prolonged the PR interval in young-aged FMT mice (*p* < 0.0001, Figure 2C). ECG waves are reflections of the myocardial cell action potentials (AP) on the body surface. AP in cardiomyocytes were known to depend on sodium, potassium, and calcium channels. Western blot analysis showed that the gut microbiota from aged mice decreased ion channel proteins expression in the ventricle (Kv4.2) and atria (Cx43, Nav1.5, Kv4.2). It also increased the levels of fibrosis-related proteins (TGF-β, α-SMA) and inflammatory cytokines (TNF-α) in the ventricle and atria of young mice (Figure 2D). In contrast, the expression of ion channel proteins and inflammatory cytokines (TNF-α) in both ventricle (CX43, Nav1.5) and atria (Cav1.2) was increased in aged-young FMT mice compared with aged-aged FMT mice (Figure 2D). Spleen weight and spleen weight-to-body weight ratio was decreased after aged microbiota FMT, and vice versa (Figure S1B), indicating that aged microbiota effected immune organs even its function. Less gap junction in intercalated discs (IDs) was found in young-aged FMT mice than young mice by immunohistochemistry (Figure 2E). These results indicated that aged gut microbiota was one of the important factors contributing to the increased susceptibility to arrhythmia in aged mice.

**Figure 3:**
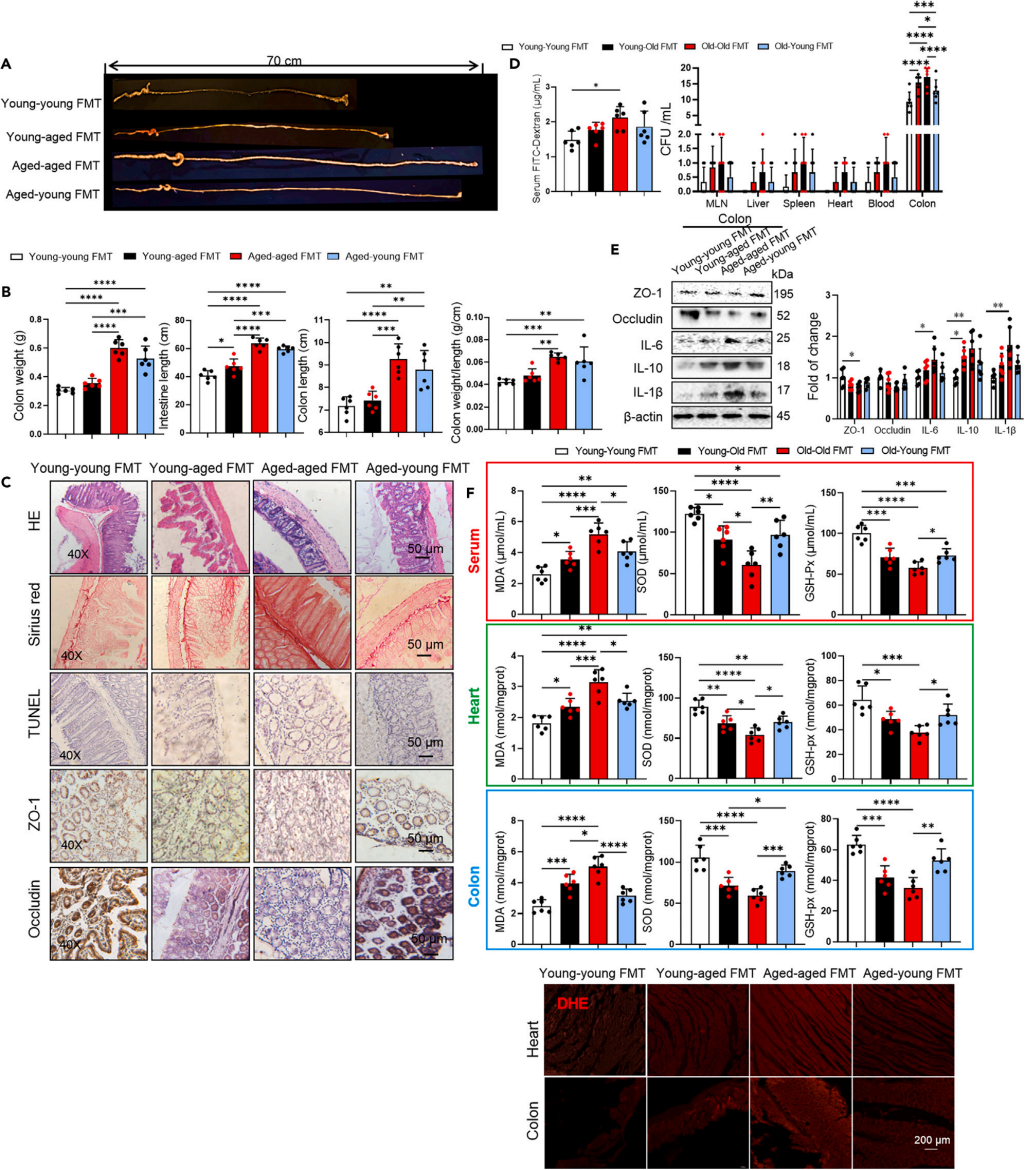
Effects of gut microbiota transplantation on gut structure and function (A) Photographs of representative images showing the intestinal of young-young FMT, young-aged FMT, aged-aged FMT, aged-young FMT mice (*n* = 6/per group). (B) Bar graphs showing the colon weight, intestinal lengths, colon lengths, colon weight/length of young-young FMT, young-aged FMT, aged-aged FMT, aged-young FMT mice (*n* = 6/per group). Data are presented as the mean ± SD. Data analyzed by one-way ANOVA with Tukey’s post-hoc test. ∗*p* < 0.05, ∗∗*p* < 0.01, ∗∗∗*p* < 0.001, ∗∗∗∗*p* < 0.0001. (C) The examples of H&E, Sirius red and TUNEL staining and representative photographs of immunohistochemistry image of ZO-1 and occludin in the colons in young-young FMT, young-aged FMT mice, aged-aged FMT mice, aged-young FMT mice (*n* = 6/per group). Scale bar, 50 μm. (D) The intestinal permeability of young-young FMT, young-aged FMT, aged-aged FMT, aged-young FMT mice measured by fluorescein isothiocyanate (FITC)-dextran in serum. The increased FITC-dextran concentration in aged-aged FMT, but not in other mice, compared to young-young FMT mice (*n* = 6/per group). Bacterial colony forming units (CFUs) of sample homogenates obtained from various tissues of different group mice were counted and quantified (*n* = 6/per group). Data are presented as the mean ± SD. Data analyzed by one-way ANOVA with Tukey’s post-hoc test or Mann-Whitney’s U test. ∗*p* < 0.05, ∗∗*p* < 0.01, ∗∗∗*p* < 0.001, ∗∗∗∗*p* < 0.0001. (E) Representative western blot and quantification of ZO-1, occludin, IL-6, IL-10, IL-1β, β-actin in colon tissue of young-young FMT, young-aged FMT, aged-aged FMT and aged-young FMT mice (*n* = 6/per group). GAPDH was used for internal normalization. Data are presented as the mean ± SD. Data analyzed by one-way ANOVA with Tukey’s post-hoc test. ∗*p* < 0.05, ∗∗*p* < 0.01, ∗∗∗*p* < 0.001, ∗∗∗∗*p* < 0.0001. (F) Cardiac, serum, colon MDA, SOD, and GSH-px levels were quantified using commercial assay kits (*n* = 6/per group). Representative pictures of DHE staining of heart and colon. Data are presented as the mean ± SD. Data analyzed by one-way ANOVA with Tukey’s post-hoc test. ∗*p* < 0.05, ∗∗*p* < 0.01, ∗∗∗*p* < 0.001, ∗∗∗∗*p* < 0.0001. DHE indicates dihydroethidium; MDA, malondialdehyde; SOD, superoxide dismutase; GSH-px, glutathione peroxidase.

Gut dysbiosis increases with age and is associated with the leakage of harmful metabolites due to damaged gut barrier function. Accumulating evidence indicates that intestinal abnormalities, including oxidative stress, microbial metabolites (such as trimethylamine N-oxide and phenylacetylglutamine), and intestinal leakage, are also important factors contributing to the role of the gut microbiota in arrhythmogenesis.^6^ Therefore, we investigated whether the structure and integrity of the intestinal barrier were altered by inter-transplantation of gut microbiota in different groups. After 6 weeks of FMT, food, water intake, fecal water content, and fecal quantity tended to decrease in young-aged mice compare to young mice, and vice versa (Figure S1C). Consistent with this, aged microbiota lengthened the small intestine (*p* = 0.0493) in young mice (Figures 3A and 3B). Analyses of H&E, Sirius red, and TUNEL staining of histological sections showed decreased fibrosis and apoptosis in aged-young FMT mice when compared with aged mice (Figure 3C). To investigate the impact of FMT on bacterial translocation, we collected tissue samples from the colon, mesenteric lymph nodes (MLN), liver, heart, and spleen. The samples were cultured on LB agar plates for 24 h and the number of colony-forming units (CFUs) was quantified (Figure 3D). The number of bacterial colonies of colon in aged mice was higher than in young mice, which reduced by young microbiota and increased by aged microbiota (Figure 3D, *p* < 0.0001). The bacterial translocation was reduced in young mice after undergoing FMT (Figure 3D, *p* < 0.0001). Consistent with this, it increased in young-aged FMT mice compared to young mice (*p* < 0.0001). To evaluate whether aged gut microbiota affects intestinal permeability, we measured serum FITC concentrations after orally administering FITC-dextran (Figure 3D). FITC concentration in serum was higher in aged mice than in young mice (*p* = 0.0181), which indicated an increased gut permeability. But only a decreasing tendency of intestinal permeability was found in aged-young FMT mice compared with aged mice (Figure 3D). These results suggested that the transplantation of gut microbiota from aged mice damaged the integrity and function of the intestinal barrier, leading to increased intestinal permeability in young mice, and vice versa. Consistent with this, no difference in localization of tight junction proteins, ZO-1 and occludin were observed on the 4 groups by immunohistochemistry (Figure 3C). Furthermore, western blot analysis showed that the gut microbiota from aged mice increased the expression of inflammatory cytokines (IL-10) in the colon (Figure 3E), and vice versa, which suggested that the aging of gut microbiota associated with intestinal barrier damage in aged mice.

Increasing studies reveal that oxidative stress contributed to aging related arrhythmia.^18^ To investigate whether the ROS is the primary mechanism behind underlying aging-related microbial dysbiosis-induced arrhythmia, we assessed the activation of ROS in the 4 groups. After 6 weeks of FMT, an elevated levels of oxidative stress markers-malondialdehyde (MDA), and dihydroethidium (DHE) staining, and reduced levels of antioxidant enzymes-superoxide dismutase (SOD) and glutathione peroxidase (GSH-px), in the serum, colon, and heart tissue was found in young-aged mice compared to young mice, and vice versa (Figure 3F), suggesting that ROS decreased cardiac ion channel protein expression associated with aging-related arrhythmia. Collectively, these results indicated that aged microbial dysbiosis increased susceptibility to arrhythmia, which associated with decreased cardiac ion channel proteins expression, intestinal barrier damage, and oxidative stress.

### Aging gut microbiota depletion decreased arrhythmia susceptibility

**Figure 4:**
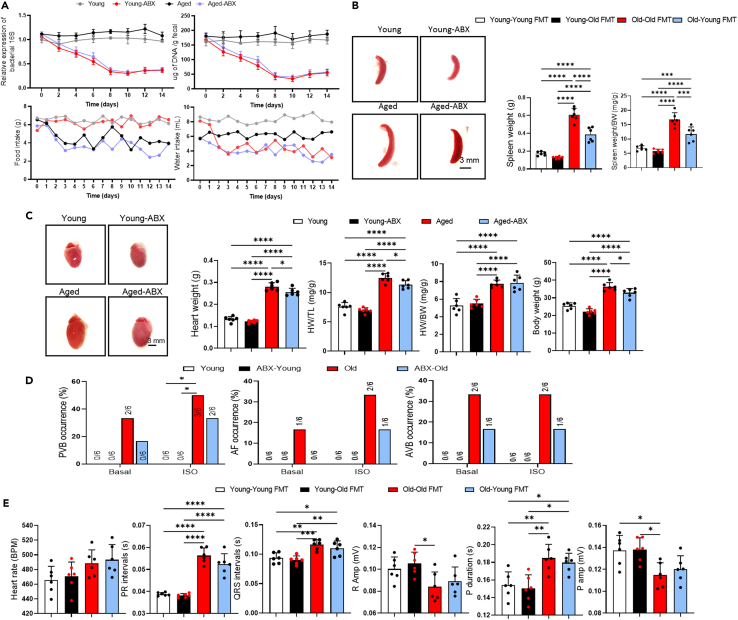
Gut microbiota deletion reduced susceptibility to arrhythmia in aged mice (A) DNA content extracted from feces of young, young-ABX, aged, aged-ABX mice. Change of bacterial load in feces from baseline, evaluated by PCR using universal bacterial 16S rRNA primers (upper line). Broken line represent average value of food and water intake amount of each group (down line) (*n* = 6/per group). (B) The overall spleen size. Scale bar, 3 mm. Quantitative analysis of spleen weight, spleen/body weight in young, young-ABX, aged, aged-ABX mice (*n* = 6/per group). Data are presented as the mean ± SD. Data analyzed by one-way ANOVA with Tukey’s post-hoc test. ∗*p* < 0.05, ∗∗*p* < 0.01, ∗∗∗*p* < 0.001, ∗∗∗∗*p* < 0.0001. (C) The overall heart size. Scale bar, 3 mm. Quantitative analysis of body weight, heart weight, heart/body weight (HW/BW), heart weight/tibia length (HW/TL) in young, young-ABX, aged, aged-ABX mice (*n* = 6/per group). (D) Differences in heart rates, PR intervals, QRS intervals, R amplitude, P duration and P amplitude among young, young-ABX, aged, aged-ABX mice (*n* = 6/per group). Data analyzed by one-way ANOVA with Tukey’s post-hoc test. ∗*p* < 0.05, ∗∗*p* < 0.01, ∗∗∗*p* < 0.001, ∗∗∗∗*p* < 0.0001. Summary of inducibility of VPB, AF, and AVB in young and aged mice. Numbers in parentheses indicate the number of mice that were induced into PVB, AF, AVB following ISO treated (*n* = 6/per group). Data analyzed by Fischer’s exact test. (E) Differences in heart rates, PR intervals, QRS intervals, R amplitude, P duration and P amplitude among young, young-ABX, aged, aged-ABX mice (*n* = 6/per group). Data analyzed by one-way ANOVA with Tukey’s post-hoc test. ∗*p* < 0.05, ∗∗*p* < 0.01, ∗∗∗*p* < 0.001, ∗∗∗∗*p* < 0.0001. ABX indicates cocktail of antibiotics.

**Figure 5:**
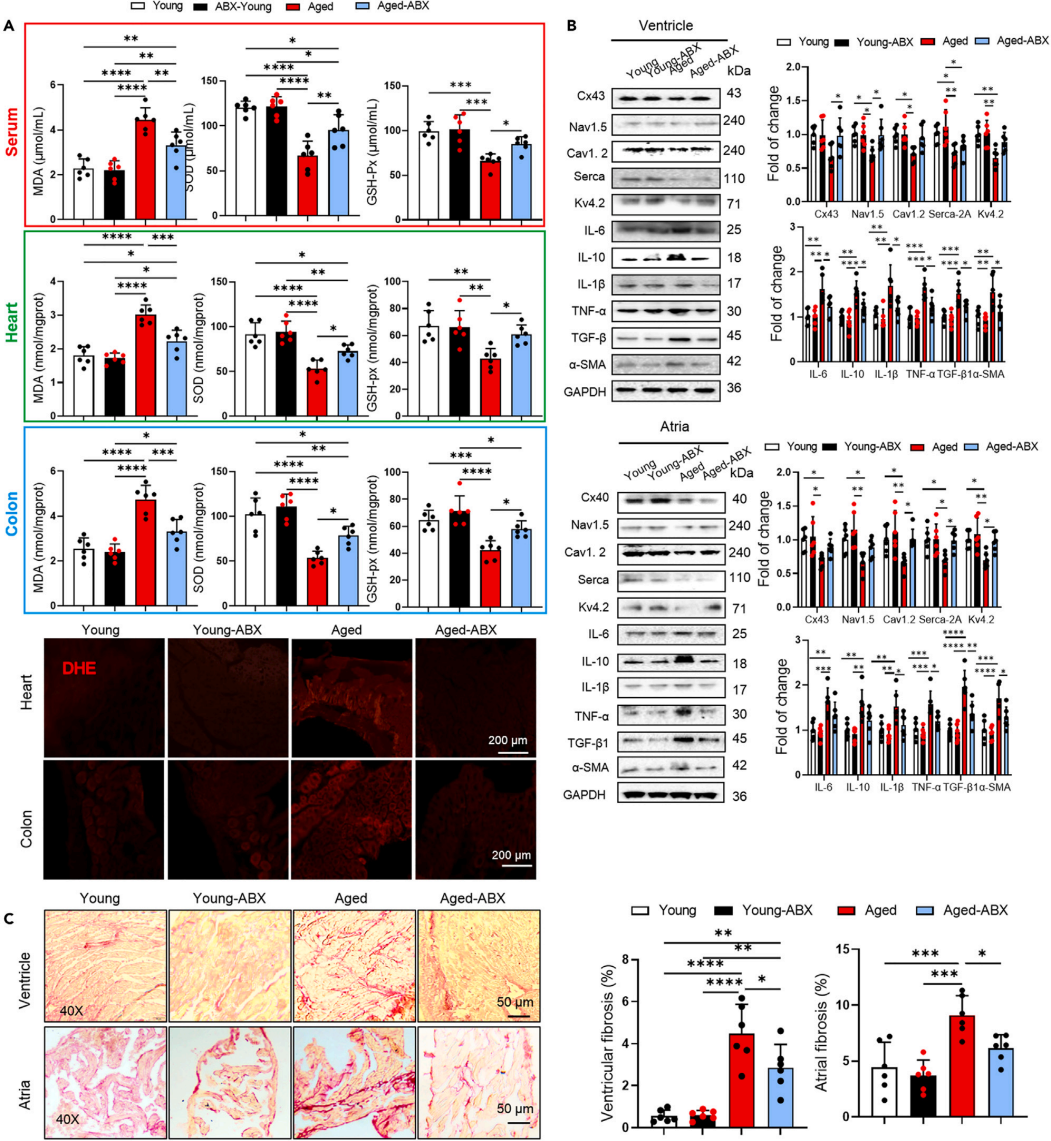
Gut microbiota deplete reduced oxidative stress (A) Cardiac, serum, colon MDA, SOD, and GSH-px levels were quantified using commercial assay kits (*n* = 6/per group). Representative pictures of DHE staining of heart and colon. Data are presented as the mean ± SD. Data analyzed by one-way ANOVA with Tukey’s post-hoc test. ∗*p* < 0.05, ∗∗*p* < 0.01, ∗∗∗*p* < 0.001, ∗∗∗∗*p* < 0.0001. (B) Representative western blot and quantification of Cx43, Cx40, Nav1.5, Cav1.2, Serca-2A, Kv4.2, IL-6, IL -10, IL -1β, TNF-α, TGF-β, α-SMA, GAPDH in ventricular and atrial tissue of young, young-ABX, aged and aged-ABX mice (*n* = 6/per group). GAPDH was used for internal normalization. Data are presented as the mean ± SD. Data analyzed by one-way ANOVA with Tukey’s post-hoc test. ∗*p* < 0.05, ∗∗*p* < 0.01, ∗∗∗*p* < 0.001, ∗∗∗∗*p* < 0.0001. (C) Representative Sirius red staining images and in the ventricular and atria (*n* = 6/per group). Scale bar, 50 μm. Gut microbiota depletion attenuated fibrosis in aged mice measured by Sirius red staining. Data are presented as the mean ± SD. Data analyzed by one-way ANOVA with Tukey’s post-hoc test. ∗*p* < 0.05, ∗∗*p* < 0.01, ∗∗∗*p* < 0.001, ∗∗∗∗*p* < 0.0001. MLN indicates mesenteric lymph node.

**Figure 6:**
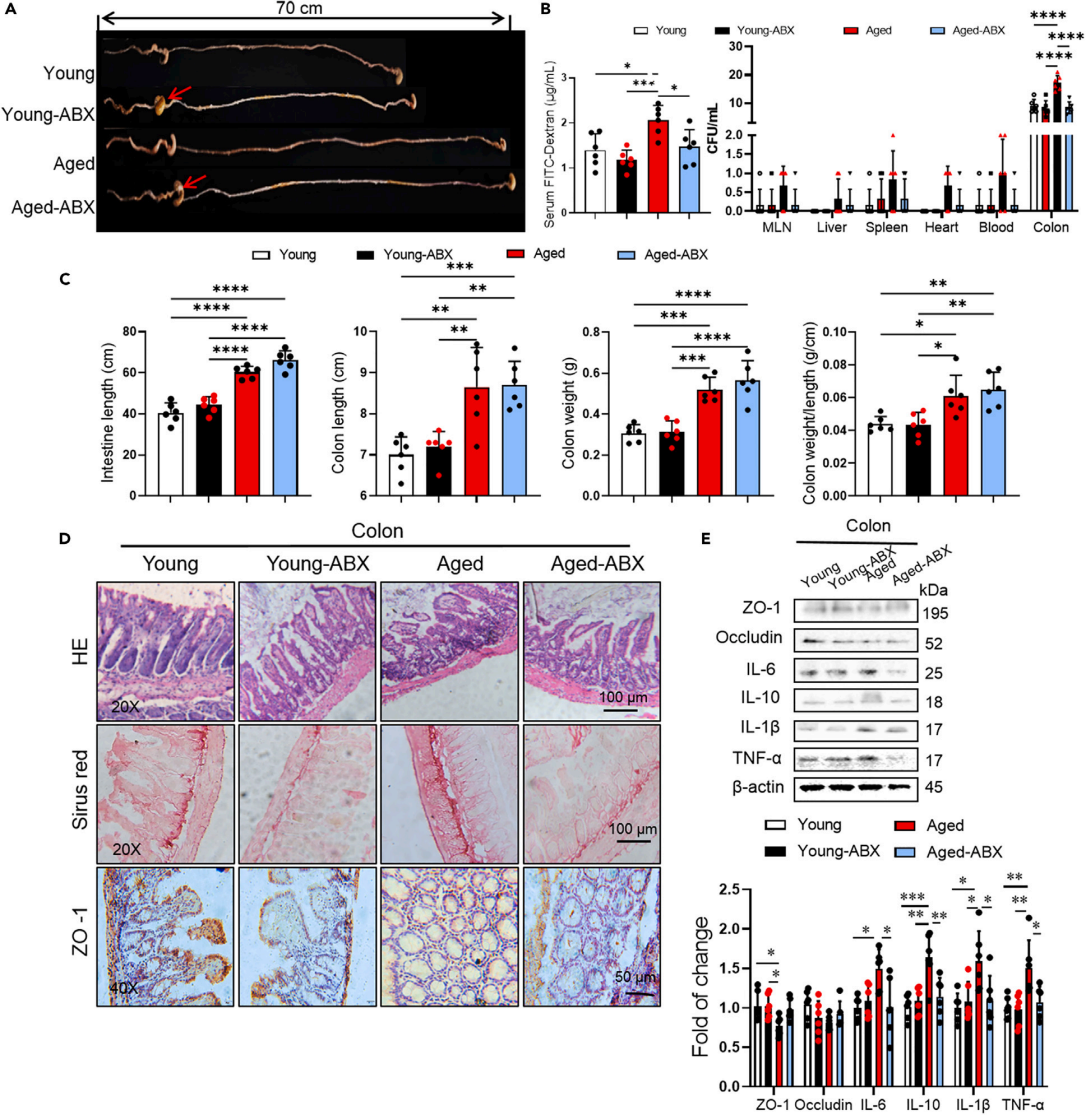
Effects of gut microbiota depletion on intestinal function (A) Photographs of representative images showing the intestinal of young, young-BAX, aged, aged-ABX mice. (B) The intestinal permeability of young, young-ABX, aged, aged-ABX mice measured by fluorescein isothiocyanate (FITC)-dextran in serum (*n* = 6/per group). The decreased FITC-dextran concentration in aged-ABX mice, compared to aged mice. Bacterial colony forming units (CFUs) of sample homogenates obtained from various tissues of different group mice were counted and quantified (*n* = 6/per group). Data are presented as the mean ± SD. Data analyzed by one-way ANOVA with Tukey’s post-hoc test or Mann-Whitney’s U test. ∗*p* < 0.05, ∗∗*p* < 0.01, ∗∗∗*p* < 0.001, ∗∗∗∗*p* < 0.0001. (C) Bar graphs showing the colon weight, intestinal lengths, colon lengths, colon weight/length of young-young FMT, young-aged FMT, aged-aged FMT, aged-young FMT mice (*n* = 6/per group). Data are presented as the mean ± SD. Data analyzed by one-way ANOVA with Tukey’s post-hoc test. ∗*p* < 0.05, ∗∗*p* < 0.01, ∗∗∗*p* < 0.001, ∗∗∗∗*p* < 0.0001. (D) The examples of HE, Sirius red staining, and representative photographs of immunohistochemistry image of ZO-1 in the colons in young, young-ABX, aged, aged-ABX mice (*n* = 6/per group). Scale bar, 50 μm. (E) Representative western blot and quantification of ZO-1, including, IL-6, IL-10, IL-1β, TNF-α, β-actin in colon tissue of young, young-ABX, aged, aged-ABX mice (*n* = 6/per group). GAPDH was used for internal normalization. Data are presented as the mean ± SD. Data analyzed by one-way ANOVA with Tukey’s post-hoc test. ∗*p* < 0.05, ∗∗*p* < 0.01, ∗∗∗*p* < 0.001, ∗∗∗∗*p* < 0.0001.

To investigate whether the aged gut microbiota enhances susceptibility to arrhythmia through oxidative stress, we depleted the gut microbiota in mice by administering antibiotic cocktails (ABX) in their drinking water for 2 weeks. Total bacterial abundance was quantified by measuring DNA content and conducting PCR amplification of the 16S RNA genes in DNA isolated from fecal bol. We examined over the course of ABX treatment, bacterial abundance began to decrease on day 4, and maintained a two-thirds reduction observed on day 8 (Figure 4A). The effectiveness of ABX treatment was demonstrated by gross morphological changes indicative of bacterial clearance, such as cecal enlargement (indicated by red arrows) and splenic atrophy (Figure 4B). A decrease in body (*p* = 0.0472), spleen (*p* < 0.0001), and heart weights (*p* = 0.0426) in aged mice after a 2-week treatment of ABX was observed, but no change in water and food intake (Figures 4A–4C). Next, we induced arrhythmia by 2 mg/kg ISO (i.p.) in the aged and ABX treated aged mice. The aged-ABX mice had a significantly lower susceptibility to PVB, AF, AVB, and ventricular tachycardia (VT) compared to the aged mice (Figure 4D). No difference was found in ECG parameters after gut microbiota deletion (Figure 4E). Taken together, these results indicated that arrhythmia susceptibility was reduced by gut microbiota-depletion. The gut microbiota depletion significantly reduced intestinal permeability and bacterial translocation from fecal to colon in aged mice. ABX treatment decreased the levels of MDA, and DHE staining, while enhancing SOD and GSH-px, in serum, colon, and heart (Figure 5A), indicating that gut microbiota depletion reduced oxidative stress. The heart weight-to-tibia length ratio (HW/TL, Figure 5C, *p* = 0.0474) was significantly lower in aged-ABX mice compared to aged mice. Furthermore, the effects of ABX on cardiac proteins were also examined. Western blot analysis showed that ABX increased ion channel proteins expression in the ventricle (Nav1.5, Cx43) and atria (Cav1.2, Serca-2A, Kv4.2) (Figures 5B and 6E). Depletion of gut microbiota in aged mice reduced fibrosis in the atria and ventricle (Figure 4F), and ABX reduced fibrosis-related proteins (TGF-β, α-SMA) levels in both the ventricle and atria, as well as inflammatory cytokines in the ventricular and colon (IL-6, IL-10, IL-1β, TNF-α), and atria (IL-6, IL-10, IL-1β, TNF-α) (Figures 5B and 6E). Altogether, these results indicated that the gut microbiota depletion reduced fibrosis and decreased cardiac ion channel proteins expression. The concentration of FITC in serum was lower in ABX treated aged mice compared to aged mice (*p* = 0.0319). In aged-ABX mice, the number of bacterial colonies in the colon was lower than aged mice (Figures 6A–6C), indicating that depletion of gut microbiota reduced intestinal permeability. Additionally, reduced intestinal fibrosis was found in aged mice (Figure 6D).

### ROS leads to arrhythmias by inhibiting ion channel proteins expression

**Figure 7:**
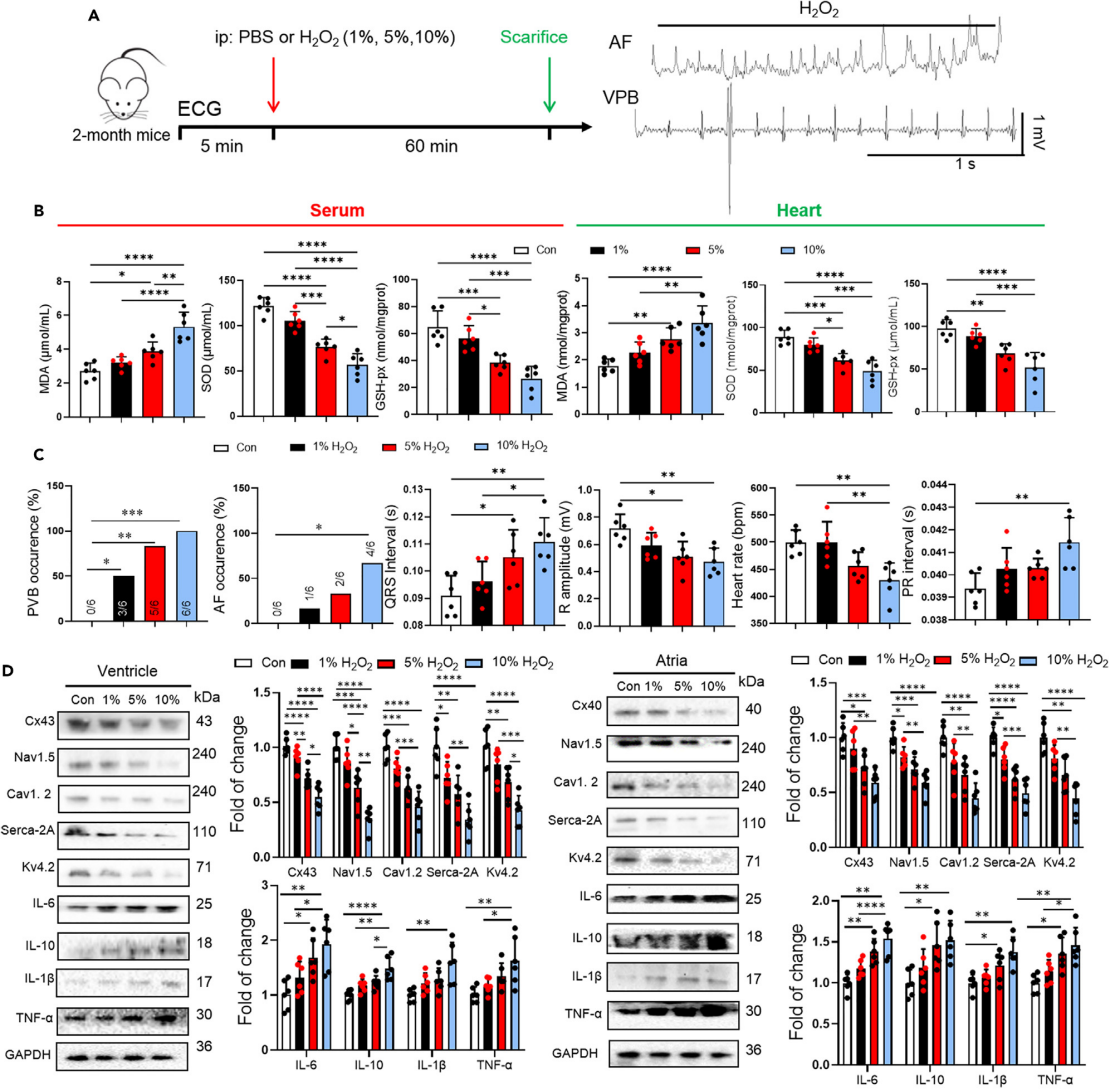
Oxidative stress promoted arrhythmogenesis (A) Experimental design for testing the effect of H_2_O_2_ (1%, 5%, 10%) treatment on mice. (B) Cardiac, serum MDA, SOD and GSH-px levels were quantified using commercial assay kits (*n* = 6/per group). Data are presented as the mean ± SD. Data analyzed by one-way ANOVA with Tukey’s post-hoc test. ∗*p* < 0.05, ∗∗*p* < 0.01, ∗∗∗*p* < 0.001, ∗∗∗∗*p* < 0.0001. (C) Summary of inducibility of VPB, AF in control and H_2_O_2_ (1%, 5%, 10%) treated mice. Numbers in parentheses indicate the number of mice with arrhythmia (*n* = 6/per group). Data analyzed by Fischer’s exact test. (D) Representative western blot and quantification of Cx43, Cx40, Nav1.5, Cav1.2, Serca-2A, Kv4.2, IL-6, IL-10, IL-1β, TNF-α, α-SMA, GAPDH in ventricular and atrial tissue of control and 1%, 5%, 10% H_2_O_2_ treatment young mice (*n* = 6/per group). GAPDH was used for internal normalization. Data are presented as the mean ± SD. Data analyzed by one-way ANOVA with Tukey’s post-hoc test. ∗*p* < 0.05, ∗∗*p* < 0.01, ∗∗∗*p* < 0.001, ∗∗∗∗*p* < 0.0001.

Accumulating evidence indicates that ROS increases with aging and play an important role in aging-related arrhythmia, which associated with decreased ion channel protein expression.^19^ Therefore, we hypothesized that ROS may promote arrhythmia by reducing the expression of atrial and ventricular ion channel proteins during aging. To investigate the role of ROS in aging-related arrhythmogenesis, young mice (8 weeks old) were intraperitoneal injected with 1%, 5%, and 10% hydrogen peroxide (H_2_O_2_) (Figure 7A). Firstly, an increased level of the oxidative stress marker—MDA and DHE staining, while decreasing the levels of SOD and GSH-px in serum, colon, and heart were found after H_2_O_2_ injection (Figure 7B). Next, oxidative stress induced arrhythmia in a concentration-dependent manner, as expected. H_2_O_2_-treated mice exhibited a high susceptibility to PVB and AF, along with a prolonged QRS duration, reduced R amplitude, decreased heart rate, and prolonged PR intervals (Figure 7C). Furthermore, the expression of ion channel protein was detected to underlying the mechanism. Western blot analysis showed that oxidative stress reduced ion channel proteins expression (Nav1.5, Cav1.2, Kv4.2, Serca-2A, Cx43, and Cx40) in atria and ventricle. It also decreased the gap junction proteins (ZO-1 and occludin) levels in colon tissue and increased the expression of inflammatory cytokines (IL-6, IL-10, IL-1β, and TNF-α) in heart and colon tissue (Figure 7D). All these results suggested that reduced ion channel protein expression in atria and ventricle led to the increased susceptibility to arrhythmia.

### Gut microbiota metabolite from aged mice enhanced ROS level in young mice

**Figure 8:**
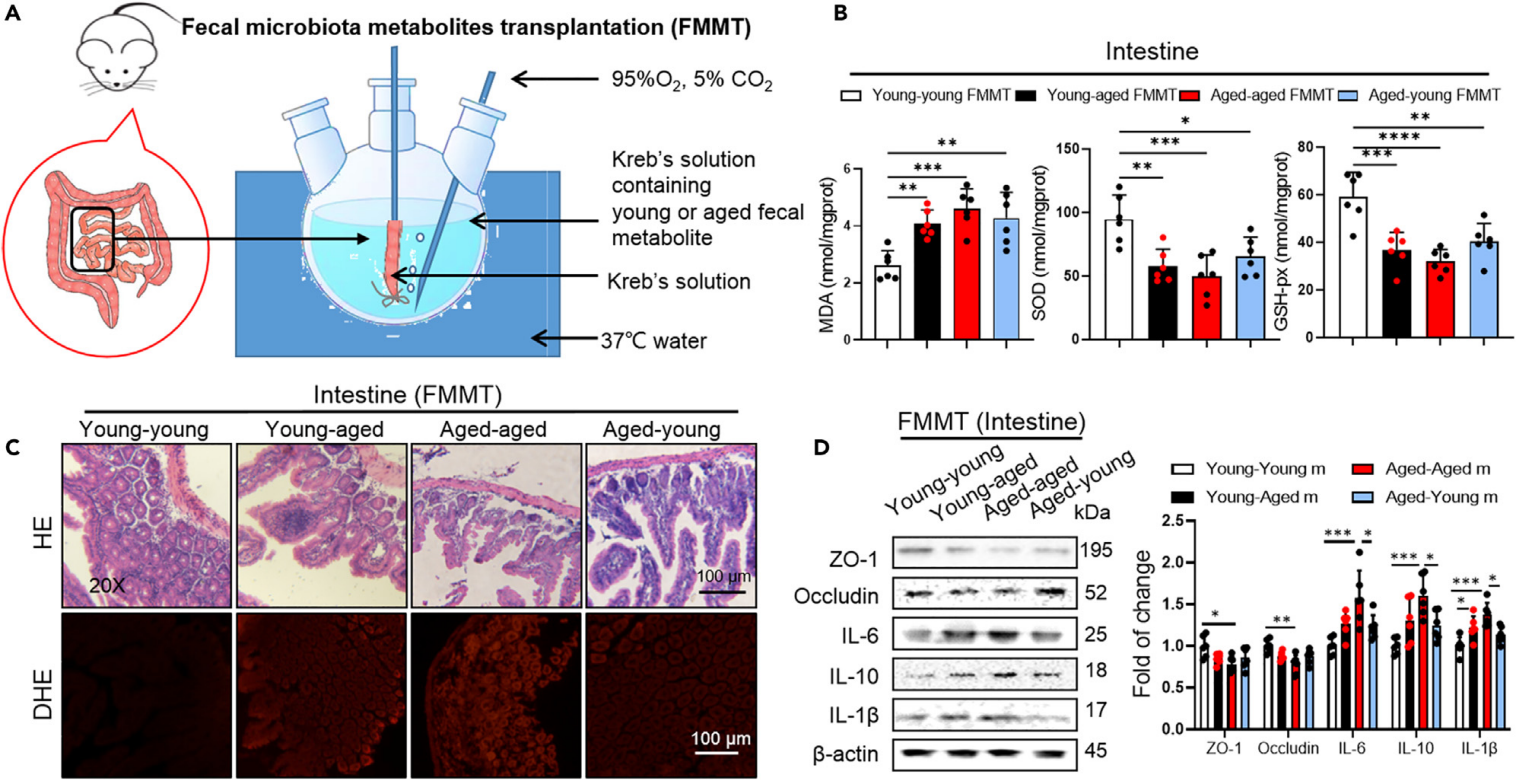
Gut microbiota metabolites from aged mice resulted in intestinal oxidative stress (A) Experimental design for testing the effect of fecal microbiota metabolites transplantation on mice. (B) Cardiac, serum, colon MDA, SOD, and GSH-px levels were quantified using commercial assay kits. (*n* = 6/per group). Data are presented as the mean ± SD. Data analyzed by one-way ANOVA with Tukey’s post-hoc test. ∗*p* < 0.05, ∗∗*p* < 0.01, ∗∗∗*p* < 0.001, ∗∗∗∗*p* < 0.0001. (C) Representative photographs of HE staining and DHE staining of colon. (D) Representative western blot and quantification of ZO-1, including, IL-6, IL-10, IL-1β, β-actin in colon tissue of young-young, young-aged, aged-aged and aged-young mice (*n* = 6/per group). Β-actin was used for internal normalization. Data are presented as the mean ± SD. Data analyzed by one-way ANOVA with Tukey’s post-hoc test. ∗*p* < 0.05, ∗∗*p* < 0.01, ∗∗∗*p* < 0.001, ∗∗∗∗*p* < 0.0001.

Redox imbalance induces oxidative injury and leads to aging-related pathologies. Accumulating evidence show that some metabolites produced by gut microbiota are directly associated with an elevation in levels of ROS,^20^ which is considered to be one of the most significant risk factors for arrhythmogenesis.^21^ To investigate what triggered colon oxidative stress, we conducted FMMT experiments on young mice by utilizing fecal metabolite samples collected from aged mice (young-aged) or young mice themselves (young-young), and performed fecal microbiota metabolite transplantation to aged mice from young mice (aged-young) or aged mice themselves (aged-aged) for 4 h (Figure 8A). We found an increased level of the oxidative stress markers—MDA and DHE, and decreased the levels of SOD and GSH-px, in serum, colon, and heart tissue were found in young-aged FMMT mice than young mice (Figure 8B). No difference of intestinal morphology was found in the 4 groups (Figure 8C). Cytokines was also affected by FMMT in colon. An increased the expression of inflammatory cytokines (IL-1β) in colon tissue found in young-aged FMMT mice than young mice (Figures 8C and 8D). A reduced inflammatory cytokines levels (IL-6, IL-10, and IL-1β) in the colon tissue was found in aged-young FMMT mice compared with aged-aged FMMT mice (Figures 8C and 8D). All the results suggested that the aged gut microbiota regulated the oxidative stress response by metabolites.

### Vitexin reduced aging-related arrhythmia susceptibility by inhibit oxidative stress

**Figure 9:**
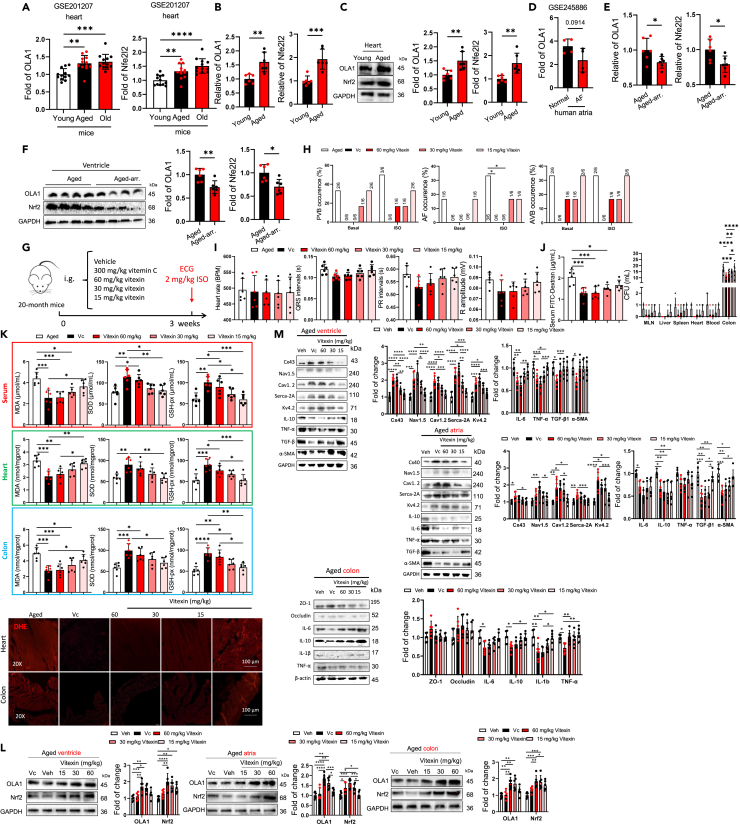
Vitexin reduced the susceptibility to arrhythmia by inhibiting oxidative stress in aged mice (A) The mRNA levels of OLA1 and Nefe2l2 in young, aged, and old mice heart from GSE dataset (GSE101207). Data are presented as the mean ± SD. Data analyzed by unpaired two tailed Student’s t test. ∗*p* < 0.05, ∗∗*p* < 0.01, ∗∗∗*p* < 0.001, ∗∗∗∗*p* < 0.0001. (B) The relative expression of OLA1 and Nefe2l2 mRNA, which was normalized by GAPDH mRNA. Data are presented as the mean ± SD. Data analyzed by unpaired two tailed Student’s t test. ∗*p* < 0.05, ∗∗*p* < 0.01, ∗∗∗*p* < 0.001, ∗∗∗∗*p* < 0.0001. (C) Representative western blot and quantification of OLA1, Nrf2, GAPDH in heart tissue of young and aged mice (*n* = 6/per group). GAPDH was used for internal normalization. Data are presented as the mean ± SD. Data analyzed by unpaired two tailed Student’s t test. ∗*p* < 0.05, ∗∗*p* < 0.01, ∗∗∗*p* < 0.001, ∗∗∗∗*p* < 0.0001. (D) The mRNA levels of OLA1 in healthy people and atrial fibrillation patient’s atria rom GSE dataset (GSE245886). Data are presented as the mean ± SD. Data analyzed by unpaired two tailed Student’s t test. ∗*p* < 0.05, ∗∗*p* < 0.01, ∗∗∗*p* < 0.001, ∗∗∗∗*p* < 0.0001. (E) The relative expression of OLA1 and Nefe2l2 mRNA in aged mice and aged mice with arrhythmia (*n* = 6/per group), which was normalized by GAPDH mRNA. Data are presented as the mean ± SD. Data analyzed by unpaired two tailed Student’s t test. ∗*p* < 0.05, ∗∗*p* < 0.01, ∗∗∗*p* < 0.001, ∗∗∗∗*p* < 0.0001. (F) Representative western blot and quantification of OLA1, Nrf2, GAPDH in heart tissue of aged mice and aged mice with arrhythmia (*n* = 6/per group). GAPDH was used for internal normalization. Data are presented as the mean ± SD. Data analyzed by unpaired two tailed Student’s t test. ∗*p* < 0.05, ∗∗*p* < 0.01, ∗∗∗*p* < 0.001, ∗∗∗∗*p* < 0.0001. (G) Protocol for administration of vitamin C, 60, 30, 15 mg/kg vitexin. (H) Summary of inducibility of VPB, AF, and AVB in vehicle, vitamin C and 60, 30, 15 mg/kg vitexin treated mice. Numbers in parentheses indicate the number of mice that were induced into VPB, AF, AVB following ISO treated (*n* = 6/per group). Data analyzed by Fischer’s exact test. (I) Differences in heart rates, P-R intervals, QRS intervals, and R amplitude among different group (*n* = 6/per group). Data are presented as the mean ± SD. Data analyzed by one-way ANOVA with Tukey’s post-hoc test. ∗*p* < 0.05, ∗∗*p* < 0.01, ∗∗∗*p* < 0.001, ∗∗∗∗*p* < 0.0001. (J) Cardiac, serum, colon MDA, SOD and GSH-px levels were quantified using commercial assay kits. (*n* = 6/per group). Representative pictures of DHE staining of heart and colon. Data are presented as the mean ± SD. Data analyzed by one-way ANOVA with Tukey’s post-hoc test. ∗*p* < 0.05, ∗∗*p* < 0.01, ∗∗∗*p* < 0.001, ∗∗∗∗*p* < 0.0001. (K) The intestinal permeability of vehicle, vitamin C and vitexin (60, 30, 15 mg/kg) treated aged mice measured by fluorescein isothiocyanate (FITC)-dextran in serum. The decreased FITC-dextran concentration in vitamin C and vitexin (60, 30, 15 mg/kg) treated mice, compared to aged mice (*n* = 6/per group). Bacterial colony forming units (CFUs) of sample homogenates obtained from various tissues of different group mice were counted and quantified (*n* = 6/per group). Data are presented as the mean ± SD. Data analyzed by one-way ANOVA with Tukey’s post-hoc test or Mann-Whitney’s U test. ∗*p* < 0.05, ∗∗*p* < 0.01, ∗∗∗*p* < 0.001, ∗∗∗∗*p* < 0.0001. (M) Representative western blot and quantification of Cx43, Cx40, Nav1.5, Cav1.2, Serca-2A, Kv4.2, IL-6, IL -10, IL -1β, TNF-α, TGF-β, α-SMA, GAPDH in ventricular and atrial, and ZO-1, occludin, IL-6, IL-10, IL-1β, β-actin in colon tissue of young-young FMT, young-aged FMT, aged-aged FMT and aged-young FMT mice (*n* = 6/per group). GAPDH was used for internal normalization. Data are presented as the mean ± SD. Data analyzed by one-way ANOVA with Tukey’s post-hoc test. ∗*p* < 0.05, ∗∗*p* < 0.01, ∗∗∗*p* < 0.001, ∗∗∗∗*p* < 0.0001. (L) Representative western blot and quantification of OLA1, Nrf2, GAPDH in ventricular, atrial, and colon tissue from mice aged treated with PBS, vitamin C, or different doses of vitexin (*n* = 6/per group). GAPDH was used for internal normalization. Data are presented as the mean ± SD. Data analyzed by one-way ANOVA with Tukey’s post-hoc test. ∗*p* < 0.05, ∗∗*p* < 0.01, ∗∗∗*p* < 0.001, ∗∗∗∗*p* < 0.0001.

We aim to find effective drugs that can treat arrhythmia in the elderly by improving oxidative stress. Recent studies have revealed that HF can result in a decrease in the expression of the oxidative stress-related protein OLA1 (Obg-like ATPase 1) in cardiac tissue, and mutations in OLA1 are also a cause of HF.^22^ Additionally, Mendelian randomization studies have also revealed a negative regulatory relationship between OLA1 and AF, indicating that reduced OLA1 expression is associated with an increased incidence of AF.^23^ We investigated OLA1 as a potential therapeutic target for arrhythmias in the elderly. Initially, we queried the GSE database to examine the regulatory relationship between OLA1 and aging, as well as the relationship between OLA1 and arrhythmias. Our results revealed that the expression of OLA1 and its downstream protein, NRF2, increased with age (Figure 9A, NCBI: GSE201207). Consistent with this, we discovered that the mRNA and protein expressions of both OLA1 and NRF2 in the heart tissue of 20-month-old mice were increased compared to 2-month-old mice (Figures 9B and 9C). Additionally, AF patients showed a non-significant decrease in OLA1 mRNA level (Figure 9D, NCBI: GSE245886). Therefore, we examined the mRNA and protein expressions of OLA1 in elderly mice with either arrhythmias or without arrhythmias. We found that in elderly mice, arrhythmias significantly reduced the mRNA and protein levels of OLA1 (Figures 9E and 9F), suggesting that OLA1 may be a potential target for regulating susceptibility to arrhythmias in the elderly.

Accumulating evidence shown that vitexin, a flavone glucoside found in numerous plant species, has been widely reported to have antioxidant and cardioprotective effects in various cardiovascular diseases, including diabetes, ischemia, and reperfusion.^24^^,^^25^^,^^26^ A study found that vitexin can specifically target for OLA1 to activate Nrf2. Therefore, to investigate whether OLA1 is involved in the occurrence of arrhythmias in the elderly and anti-oxidative role, a dose of 15, 30, and 60 mg/kg vitexin (OLA1 activator) was orally administered to aged mice, and 300 mg/kg of vitamin C was used as a positive drug (Figure 9G). After 2-week treatment, we observed that inducibility of PVB, AF, and AVB of aged mice were decreased by vitexin (Figure 9H). No difference was found in ECG parameters among the 4 groups (Figure 9I). Meanwhile, ISO rarely induced arrhythmia when treated with vitexin, whereas it commonly induced arrhythmia in aged mice. Vitexin decreased the levels of oxidative stress markers, such as MDA and DHE, and increased the activity of antioxidant enzymes, such as SOD and GSH-px, in the serum, colon, and heart tissue in aged mice (Figure 9K). Next, decreased intestinal permeability and bacterial translocation from fecal to colon in aged mice was in a concentration-dependent manner (Figure 9J). Further, western blot analysis showed that downregulated expression of atrial and ventricular ion channel proteins (Nav1.5, Cav1.2, Kv4.2, Serca-2A, Cx43, and Cx40), and upregulated expression of heart and colon tissue inflammatory cytokines (IL-6, IL-10, TNF-α) and fibrosis protein (TGF-β, α-SMA) in aged mice were significantly reversed by vetixin and vitamin C (Figure 9M), implying that the vitexin significantly reduced ROS and increased atrial and ventricular ion channel proteins expression in aged mice. And vitamin C, showed excellent antioxidant and therapeutic effects. In order to study whether vitexin can resist aging, we detected the expression of aging-related proteins using western blot. We found that 60 mg/kg vitexin reduced the expression of p21 and p53, and pro-apoptotic protein-bax, while increased the expression of apoptosis-inhibiting protein-bcl-2 (Figure S2B). In addition, 60 mg/kg vitexin also improved heart aging by reducing the levels of creatine kinase-MB (CK-MB) and cardiac troponin I (cTnI) (Figure S2C), suggesting that 60 mg/kg vitexin has a slight improvement on aging. Altogether, these results indicated that vitexin and vitamin C in intestine decreased arrhythmia susceptibility.

**Figure 10:**
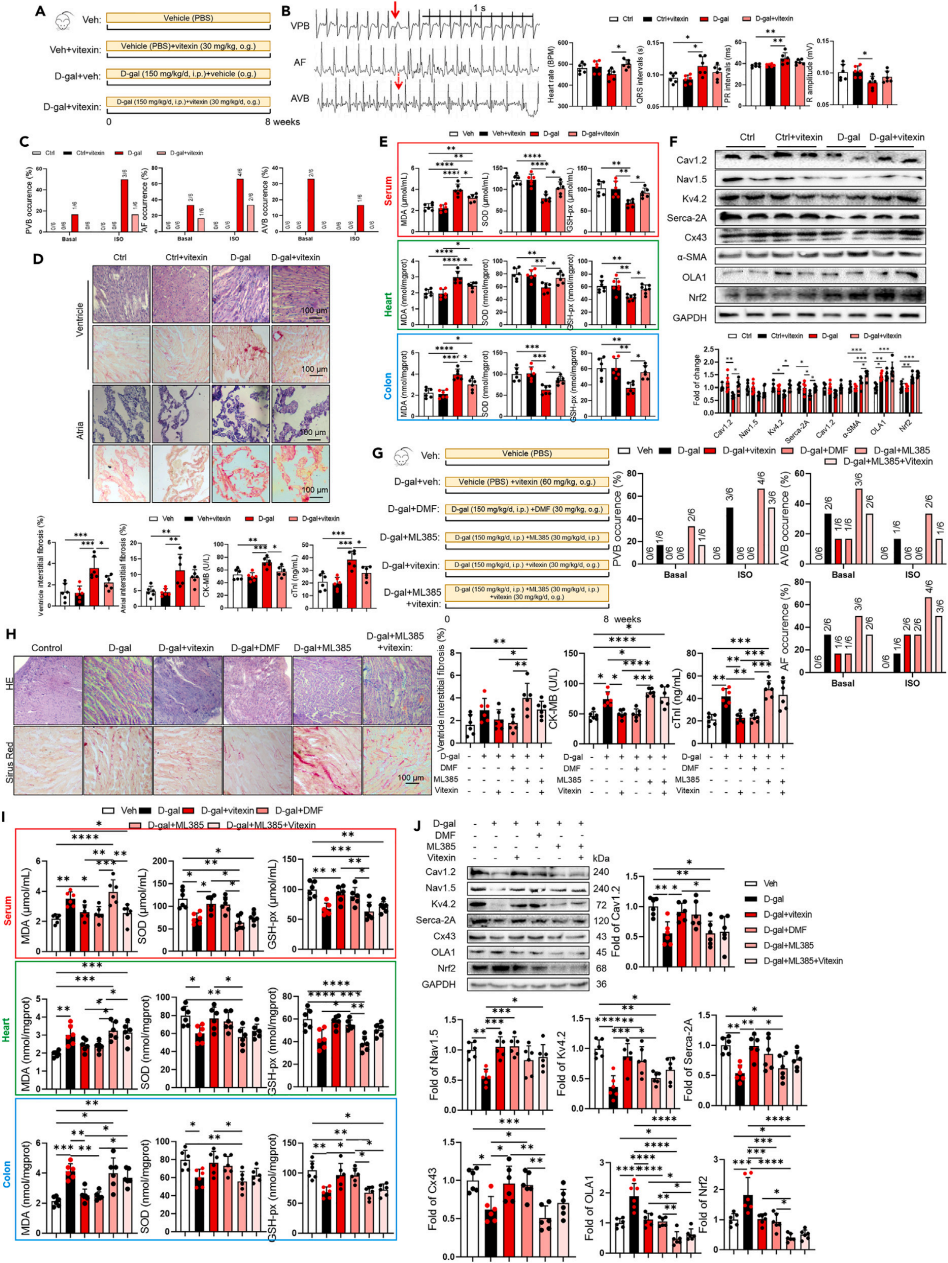
Vitexin improved aged related arrhythmia through OLA1-Nrf2 signaling pathway (A) Experimental design for testing the anti-aging effect of vitexin on aged mice induced by 150 mg kg D-gal. (B) Sample ECG traces of three different types of arrhythmic events: ventricular premature beats (VPB), atrial fibrillation (AF), and atrioventricular block (AVB). (C) Summary of inducibility of VPB, AF, and AVB in vehicle, and vitexin treated aged mice. Numbers in parentheses indicate the number of mice that were induced into VPB, AF, AVB following ISO treated (*n* = 6/per group). Data analyzed by Fischer’s exact test. And differences in heart rates, P-R intervals, QRS intervals, and R amplitude among different group (*n* = 6/per group). Data are presented as the mean ± SD. Data analyzed by one-way ANOVA with Tukey’s post-hoc test. ∗*p* < 0.05, ∗∗*p* < 0.01, ∗∗∗*p* < 0.001, ∗∗∗∗*p* < 0.0001. (D) The examples of HE, Sirius red staining in the ventricle and atria from different group of mice (*n* = 6/per group). Scale bar, 100 μm. Viteixn attenuated fibrosis in aged mice measured by Sirius red staining. Data are presented as the mean ± SD. Data analyzed by one-way ANOVA with Tukey’s post-hoc test. ∗*p* < 0.05, ∗∗*p* < 0.01, ∗∗∗*p* < 0.001, ∗∗∗∗*p* < 0.0001. (E and I) Cardiac, serum, colon MDA, SOD and GSH-px levels were quantified using commercial assay kits. (*n* = 6/per group). Data are presented as the mean ± SD. Data analyzed by one-way ANOVA with Tukey’s post-hoc test. ∗*p* < 0.05, ∗∗*p* < 0.01, ∗∗∗*p* < 0.001, ∗∗∗∗*p* < 0.0001. (F) Representative western blot and quantification of Cx43, Nav1.5, Cav1.2, Serca-2A, Kv4.2, α-SMA, OLA1, Nrf2, GAPDH in ventricle and atria in the four groups (*n* = 6/per group). GAPDH was used for internal normalization. Data are presented as the mean ± SD. Data analyzed by one-way ANOVA with Tukey’s post-hoc test. ∗*p* < 0.05, ∗∗*p* < 0.01, ∗∗∗*p* < 0.001, ∗∗∗∗*p* < 0.0001. (G) Experimental design for testing the anti-aging effect of Nrf2 activator and inhibitor on aged mice induced by 150 mg kg D-gal. Summary of inducibility of VPB, AF, and AVB in vitexin, DMF, ML385 treated aged mice. Numbers in parentheses indicate the number of mice that were induced into VPB, AF, AVB following ISO treated (*n* = 6/per group). Data analyzed by Fischer’s exact test. (H) The examples of HE, Sirius red staining in the ventricle from different group of mice (*n* = 6/per group). Scale bar, 100 μm. Viteixn and DMF attenuated fibrosis in aged mice measured by Sirius red staining. Data analyzed by one-way ANOVA with Tukey’s post-hoc test. Serum levels were quantified using commercial assay kits. (*n* = 6/per group). Data are presented as the mean ± SD. Data analyzed by one-way ANOVA with Tukey’s post-hoc test. ∗*p* < 0.05, ∗∗*p* < 0.01, ∗∗∗*p* < 0.001, ∗∗∗∗*p* < 0.0001. (J) Representative western blot and quantification of Cx43, Nav1.5, Cav1.2, Serca-2A, Kv4.2, α-SMA, OLA1, Nrf2, and GAPDH in ventricle in the four groups (*n* = 6/per group). GAPDH was used for internal normalization. Data are presented as the mean ± SD. Data analyzed by one-way ANOVA with Tukey’s post-hoc test. ∗*p* < 0.05, ∗∗*p* < 0.01, ∗∗∗*p* < 0.001, ∗∗∗∗*p* < 0.0001.

To further investigate the impact of vitexin and its target OLA1 on aging and the susceptibility to arrhythmia in the elderly, we have established an acute aging mouse model through the administration of D-galactose (intraperitoneal injection at 150 mg/kg/day) for 56 days (Figure 10A). Vitexin-treated mice were gavaged with 30 mg/kg/day vitexin, and the results showed that after 56 days of D-galactose injection, vitexin reduced the incidence of PVB, AF, and AVB in both basal and 2 mg/kg ISO stimulation (intraperitoneal injection) condition (Figure 10B), but could not improve the prolongation of QRS and PR intervals caused by D-gal (Figure 10C). At the same time, vitexin restored the increased atrial and ventricular fibrosis caused by D-gal and reduced the levels of cardiac damage markers (CK-MB, cTnI) (Figure 10D). Most importantly, vitexin decreased the cardiac, serum, and intestinal oxidative stress markers (MDA) and increased the level of antioxidant enzymes (SOD, GSH-px) (Figure 10E). We found that vitexin significantly increased the expression of OLA1 and Nrf2 in heart and intestine using western blot (Figure 10F), thus we need further elucidated whether OLA1-Nrf2 signaling pathway is one of the mechanism2 for anti-aging effect of vitexin.

In order to further verify the role of the OLA1-Nrf2 signaling pathway in the anti-aging effect of vitexin, we administered experiment aging mice (induced with D-gal) with vitexin (30 mg/kg), Nrf2 agonist-DMF (30 mg/kg), or Nrf2 inhibitor-ML385 (30 mg/kg), or combination of ML385 and vitexin, as demonstrated in Figure 10G. We found that the activation of Nrf2 by DMF exhibited an anti-aging effect similar to that of vitexin. Specifically, DMF reduced the incidence of PVB, AVB, and AF in basal condition and after ISO stimulation (Figure 10G), cardiac fibrosis and enhanced cardiac damage markers (CK-MB, cTnI) (Figure 10H), and reversed the reduction in the expression of cardiac ion channel proteins caused by D-gal (Figure 10J). Importantly, DMF reversed the increased levels of oxidative stress markers MDA, and restored the reduced antioxidant enzymes SOD and GSH-px in heart, serum, and intestines (Figure 10I), which were caused by D-galactose exposure. However, administration of the Nrf2 inhibitor ML385 exacerbated the previous aging phenotypes. Furthermore, when vitexin and NRF2 inhibitor ML385 were given simultaneously, it blocked the anti-aging effect of vitexin and did not exhibit the protective effect against aging. In summary, vitexin can reverse D-galactose-induced aging in mice by activating the OLA1-Nrf2 signaling pathway to achieve antioxidant effects.

## Discussion

In the current work, we demonstrated that aged gut microbiota exerts arrhythmia susceptibility in both aged mice and young-aged FMT mice. The underlying mechanism include intestinal barrier dysfunction, elevated ROS levels, decreased cardiac ion channel proteins expression, and increased activities of inflammatory cytokines. Moreover, vitexin and vitamin C prevents β-adrenergic-induced arrhythmia in aged mice. Thus, this study provides insights into the pathophysiological relationship between aging gut microbiota and susceptibility to arrhythmia. It opens up an avenue for therapeutic strategies to treat age-related arrhythmogenesis.

Research has shown that the number of intestinal microorganisms increases with age, and the microbiota of the elderly has more individual differences with a different composition compared to young people. The study found that the ratio of Firmicutes to Bacteroidetes (F/B) is lower in the elderly compared to young people, and there is a decrease in species that produce SCFAs (especially butyrate), such as the Clostridium cluster, reflecting poor adaptive remodeling of the intestinal microbiota as age increases. Further research has revealed that a lower relative abundance of Bacteroidetes and a higher relative abundance of Firmicutes can affect energy balance in animals and humans.^4^^,^^5^^,^^6^^,^^7^^,^^8^^,^^9^^,^^10^^,^^11^

FMT is a procedure to transplant the gut microbiota of a healthy donor into the gastrointestinal tract of a recipient with gut dysbiosis.^27^ FMT significantly improved natural aging-related cardiovascular diseases, central nervous system (CNS) diseases, tumor by improving glucose sensitivity, inflammaging, antioxidative capacity, and intestinal barrier.^28^^,^^29^^,^^30^^,^^31^ In our study, we demonstrated that transplantation of young gut microbiota into aged mice ameliorated multiple organs aging, including the liver, spleen, lung, kidney, brain, and heart (Figure S1A). FMT has been widely reported, with Zhang Y et al. reporting the dysbiosis of aged gut microbiota leads to elevated LPS and impaired glucose tolerance, which promotes aging-related AF by activating the atrial NLRP3-inflammasome, and the long-term intervention with a healthy young microbial population can prevent aging-related AF.^6^ Here, it was found that gut microbiota in old mice increased AF susceptibility in young mice and vice versa. However, the AF reported by Zhang Y et al.,^6^ was induced by programmed electrical stimulation (PES), whereas ours was induced by 2 mg/kg ISO with a lower incidence of AF. ISO injection and PES are usually used to induce arrhythmias, and PES induced more. The manifestations of acquired arrhythmia include AF, PVB, AVB, and VT. Although the inductivity of PES is higher, we believe that ISO could better display physiological stimuli in patients or natural elderly. After all, there is ISO stimulation under physiological conditions but no PES.

Although the aged gut microbiota contributes to arrhythmia in young mice, its incidence was lower than that in aged mice, indicating that the arrhythmia in aged mice did not only come from gut microbiota. Nevertheless, it reduced incidence of ISO-induced arrhythmias, the effect of young gut microbiota transplantation was mildly. We agree that the major cause of aging-related arrhythmias is senescence. Downregulated expression of ion channel proteins has been shown to be associated with impaired cardiac electrophysiology and aging-related arrhythmias in animal models and clinical studies.^11^^,^^32^ A variety of ion channels were altered in the heart of aged mice. Similarly, here, although the microbiota regulated ion channel proteins levels, overall, downregulated cardiac ion channel proteins levels in aged heart were more likely to be caused by senescence. For example, the expression of connexins, and sodium, potassium and calcium channel proteins markedly decreases with aging, which are all factors affecting the cardiac action potential (AP). Nav1.5 channels mediate inward sodium currents (INa) and induce fast depolarization to initiate an intracellular excitation-contraction coupling cascade. Nav1.5-mediated INa can be divided into peak sodium current (INa-p) and late sodium current (INa-l). *SCN5A* mutations can impair Nav1.5 function, alter the amplitude and duration of INa-P and INa-L, and thus cause different types of lethal arrhythmias, including Brudaga syndrome, long QT syndrome, cardiac conduction disease, sick sinus syndrome, AF, progressive cardiac conduction defect, dilated cardiomyopathy heart disease, and multifocal purkinje-associated premature contractions.^33^ Cav1.2 channels play a crucial role in cardiac electrophysiological activity, providing an important substrate for cardiac electrophysiological activity. Cav1.2 channels participate in excitation-contraction (EC) coupling via L-type calcium current (ICaL), control action potential duration (APD), and regulate cardiomyocyte gene expression, thus playing an important role in cardiac function. It is responsible for EC coupling, control of APD and regulation of gene expression in cardiomyocytes. Any small change in Ca2 current (ICaL) can lead to a fatal arrhythmia event.^34^ The fast transient outward potassium current (Ito) is formed by a voltage-gated α pore-forming subunit (Kv4.2 and Kv4.3 in mice) and the accessory β subunit KCHIP2. I-to is an outward potassium current that can slow down the repolarization of action potential 1 phase and affect APD. When Kv4.2 and Kv4.3 levels downregulated, the density of Ito was decreased in aged cardiomyocytes, which leads to abnormal repolarization and prolonged APD, which can lead to fatal ventricular arrhythmias.^35^

It was known that the alteration of Na^+^, k^+^, and Ca^2+^ channel proteins is involved in many kinds of arrhythmias, such as PVB after Nav1.5 knockouts. In our study, the expression of ion channel proteins also changed. We demonstrated the relationship between ion channel protein level and gut microbiota homeostasis in old mice, but the FMT of the young microbiota did not completely eliminate the ion channel protein alteration, which suggested that the aged microbiota was only a reason for downregulated expression of cardiac ion channel protein during aging, just as the young microbiota transplantation couldn’t eliminate ISO-induced arrhythmia in aged completely. The interaction between microbiota dysbiosis and the gut plays a vital role in the development of aging-related diseases. The inflammatory cascade caused by arrhythmias has traditionally been considered aseptic inflammation. Although important studies in recent years revealed a potential link between gut bacterial translocation and myocardial ischemia/reperfusion injury, convincing evidence is still lacking.^36^ Although the intestinal bacterial translocation of aged mice increased, no direct relationship with aging-related arrhythmia. Age-related microbial dysbiosis led to increased intestinal permeability, which facilitates the transfer of harmful substances and pathogens from gut to blood. These results suggest that age-related gut dysbiosis may lead to significant pro-inflammatory effects and aggravate the intestinal inflammatory response, which could explain the increased intestinal permeability. More importantly, western blot analysis found that the levels of inflammatory cytokines were upregulated in the colon of young healthy mice that received the aged microbiota. Although the expression of cardiac ion channel protein and intestinal tight junction protein decreased with aging, the increase in inflammatory factors was very slight. Consistent evidence was also observed in western blot results of this study. Many data from FMT, gut microbiota depletion assay, gut microbiota metabolites transplantation, and vitexin treatment did not show statistically significant results, just an increasing or decreasing trend.

Generally speaking, oxidative stress is one of the main cause that trigger aging, and chronic oxidative stress is one of the characteristics of aging and involved in the development of aging-related disease.^4^^,^^5^^,^^6^^,^^7^^,^^8^^,^^9^ The damage to antioxidant function caused by aging is one of the reasons for oxidative stress, and it is usually related to the aging of gut microbiota.^37^^,^^38^ Studies have shown that there is a relationship between intestinal flora and the antioxidant function in aging animals.^39^^,^^40^ Cardiac cells are post-mitotic cells, which are more prone to oxidative stress than mitotic cells. The relief of oxidative stress in aging cells is related to the restoration of intestinal flora structure.^41^ As an organ that requires high energy produced by mitochondrial respiration, the heart is particularly vulnerable to oxidative stress. In addition, deleting intestinal flora in mice by antibiotics can reduce age-related vascular oxidative stress and restore antioxidant enzymes, which is similar to our research. After using mixed antibiotics cocktail (ABX) to clear intestinal flora, the level of oxidative stress in heart reduced and the activity of antioxidant enzymes increased. So far, there are few studies on the direct impact of aging intestinal flora on the host’s antioxidant defense. By transplanting fecal microbiota (FMT) to colonize young germ-free rats’ intestinal flora with old rats, it found that the intestinal flora of old rats decreased superoxide dismutase (SOD) activity and an increased malondialdehyde (MDA) level in serum.^42^ Overall, aging intestinal flora may mediate oxidative stress in the host by damaging antioxidant defense. Research has shown that some metabolites of intestinal bacteria (small formylated peptides and LPS), can trigger the production of ROS,^43^^,^^44^ and all these metabolites increase with aging.^45^ However, the impact of the aging intestinal flora on the production of ROS still need further research. The intestinal flora is a critical metabolic organ, and its composition and quantity vary with age. FMT can colonize the gut with microbiota from another individual, and it has been demonstrated that young gut microbiota can indeed ameliorate the functional decline associated with aging. Young mice exhibit low levels of ROS, which are also regulated by their intestinal microbiota. While vitexin has been shown to improve cardiac and intestinal aging, we still cannot definitively determine whether its target site is within the microbiota or within the organism. We have only validated the effect of FMT and vitexin on improving arrhythmias by reducing oxidative stress, but it is evident that their therapeutic benefits in this regard extend beyond this single mechanism.

More importantly, we also found that oxidative stress increased with aging. Additionally, it has been reported that oxidative stress produced by intestinal flora promoted arrhythmias. For example, studies have shown that indolenol sulfate, a harmful metabolite produced by pathogens such as Salmonella, promoted myocardial fibrosis and atrial matrix assembly. It also affects the spontaneous activity of the sinoatrial node through oxidative stress.^11^^,^^46^^,^^47^ Previous studies have focused on AF, and our study provides valuable insights into ventricular arrhythmias from a perspective. We explored the interaction between gut microbiota metabolites and cardiac ion channels, revealing a direct interaction between gut microbiota metabolites and oxidative stress. Transplantation of aged gut microbiota successfully transmits ROS from the elderly to young healthy recipient mice. *In vitro*, the metabolites produced by the gut microbiota of aged mice can also induce oxidative stress in the young intestine. These findings confirm the spread of oxidative stress among the gut microbiota-metabolism-colon, leading to decreased cardiac ion channel proteins expression and increased susceptibility to arrhythmia. It could be speculated that if the dysbiosis-related arrhythmogenesis in the aged microbiota is dependent on downregulated expression of ion channel proteins, there would be inducers. Using both ROS detection and western blot, we found that both aged the gut microbiota and H_2_O_2_ treatment reduced cardiac ion channel proteins expression and activated inflammatory cytokines, resulting in cardiac electrophysiological disorders. Consistent evidence was also observed in gut microbiota-induced cardiac oxidative stress and downregulated expression of cardiac ion channel in aged mice increased susceptibility to arrhythmias. Inflammation is associated with increased ROS and induces oxidative damage to a variety of biomolecules, leading to protein dysfunction, genetic instability, or cell death. Additionally, oxidative stress adversely affects ion homeostasis by altering the structure and electrical function of cardiac ion channels. The depolarization induced by H_2_O_2_ increased the late sodium current (I), transient outward potassium current and L-type calcium current.^48^ H_2_O_2_ increased ventricular myocytes INa-p in a concentration-dependent manner, which may be related to the direct oxidation of cell membranes.^49^ H_2_O_2_ reduces Ca current (ICa) amplitude, [Ca2]i transients, and active cells shorten. H_2_O_2_ prolongs the relaxation period of the [Ca2]i transient and activates the outgoing membrane current consistent with the ATP-sensitive K current (IK, ATP), but does not change the voltage dependence of ICa, shortening the peak of the [Ca2]i transient or active cell. After exposure to H_2_O_2_, ICa induced smaller [Ca2]i transients than under controlled conditions. H_2_O_2_ interferes with EC coupling of guinea pig cardiomyocytes by damaging ICa, activating IK and ATP. However, H_2_O_2_ stimulates Na-Ca exchange and reduces Serca2 storage. Additionally, diastolic [Ca2]i is elevated when cardiomyocytes are still in an excited state.^50^ This phenomenon appears to play a critical role in triggering various types of ventricular arrhythmias. *In vitro*, fecal microbial metabolites transplantation was used to detect the source of intestinal oxidative stress confirmed that colonic oxidative stress was regulated by fecal microbial metabolism in aged mice. Similar results have been reported for the ability of Lactobacillus and Bifidobacterium to convert nitrate and nitrite into nitric oxide (NO), making the intestinal epithelium a rich source of NO. Similarly, Streptococcus and Bacilli also use NOS to produce NO from L-arginine.^51^ High concentrations of NO produce ROS and nitrogen (RONS), such as superoxide and H_2_O_2_, which are associated with myocardial fibrosis, inflammation, and ion channel dysfunction. It has been reported that the total antioxidant capacity of the colon is lower than that of the small intestine.^52^ Indeed, one of the major limitations of this study is that we used the colon in both FMT and gut microbiota deletion to detect oxidative stress from the gut microbiota. However, when we transplanted fecal microbiota metabolites *in vitro*, we deviated from the design by using the small intestine of mice. In this study, we chose the small intestine containing soft content because the presence of colonic content makes the intestinal tissue difficult to valgus. In FMT and intestinal microflora deletion (ABX) mice, only colon was used to detect oxidative stress, and the relationship between microflora and oxidative stress was studied. The choice was made because levels of oxidative stress are higher in the colon than in the small intestine, and stool samples could only come from the *ex vivo* colon.

We investigated the susceptibility to arrhythmia in aged mice after blocking oxidative stress with vitexin and vitamin C. Our findings indicated that vitexin effectively reduced the levels of ROS, preserved the activity of antioxidant enzymes, and suppressed both arrhythmia susceptibility and the production of pro-inflammatory cytokines in aged mice. Vitexin has been reported to possess numerous properties, such as anti-inflammatory, antimicrobial, and antioxidant, which make it an ideal ingredient for therapies targeting cardiac disease. Vitexin inhibits the IIS pathway by occupying the adenosine-triphosphate binding site pocket of IGFR, which in turn reduces IGFR expression and promotes longevity and fitness.^53^ The anti-aging effect of vitexin was also confirmed in H&E staining of the heart, intestine, liver, spleen, lung, and kidney (Figure S2A). Previous studies have shown that vitexin increased cell viability and reduced tissue damage by increasing cell resistance to oxidative stress-inducing agents.^54^^,^^55^^,^^56^ Meanwhile, the anti-inflammatory activity of vitexin has attracted more and more attention, and has been studied extensively. Vitexin downregulated the release of inflammatory cytokines (TNF-α, IL-1β, and IL-6) and enzymes (iNOS) by regulating transcription factors and kinases (Nrf-2, NF-κB, and MAPKs),^57^^,^^58^ which consistent with our work. Moreover, vitexin has myocardial protective effects on chronic myocardium ischemia rats by inhibiting cardiomyocyte apoptosis and lipid peroxidation.^59^ In summary, these results indicated that vitexin had proven effectiveness on anti-oxidation.

OLA1, a member of the GTPase protein family, possesses both GTPase and ATPase activities, with higher ATP binding and hydrolysis efficiency. OLA1 is involved in physiological and pathological processes such as intercellular matrix adhesion, epithelial-mesenchymal transition, spindle assembly, and oxidative stress.^60^^,^^61^ Studies have shown that OLA1 serves as a negative regulator of intracellular antioxidant responses, primarily influencing cellular antioxidant capacity by modulating the dynamic balance of reduced thiols. OLA1 plays a critical role in cardiac structure and function, and OLA1 knockout mice exhibit dilated cardiomyopathy and dysfunction in the heart through the activation of the GSK-beta/beta-catenin signaling pathway.^62^ Previous studies have shown that the interaction between the OLA1-vitexin complex and the Kelch-like ECH-associated protein 1 (Keap1) protein disrupts the Keap1-Nrf2 interaction, which in turn activates Nrf2, ultimately exhibiting an anti-inflammatory effect.^63^ We have also discovered similar results, in which veteixn activated the OLA1-NRF2 pathway. When Nrf2 was inhibited, the antioxidant effect of vitexin was blocked. Conversely, when NRF2 was activated, it produced an antioxidant effect similar to that of vitexin, even though Nrf2 is inherently an antioxidant protein. Nuclear factor erythroid 2-related factor 2 (Nrf2) is involved in various physiological and pathological processes such as metabolism, inflammation, and oxidative stress. The regulation of Nrf2 activity mainly depends on its protein-protein interaction with Keap1. In normal, Nrf2 degraded after binding with Keap1. During oxidative stress, Nrf2 fails to bind with Keap1, resulting in a feedback activation of Nrf2 and increased its transcription.^64^

Among all organs, the heart is the most vulnerable to premature aging and free radical oxidative stress. Clinical studies have clarified the role of free radical damage and cardiovascular diseases. Although it reacts quickly and is thus vulnerable to oxidative stress, the heart can also benefit from some plant nutrients, antioxidants, and nutrients, such as vitamin C, E, and beta carotene, which have strong antioxidant effects. Multiple antioxidants can improve intestinal flora, such as vitamin C and curcumin, which also have an anti-arrhythmic effect. This suggests that regulating arrhythmias by improving intestinal flora and oxidative stress has a certain theoretical basis. Vitamin C is a powerful antioxidant that helps protect cells from oxidative damage. It also plays an important role in inflammation regulation, and many other processes that are crucial for healthy aging. In addition, antioxidant drug TEMPOL promotes functional metabolic changes in intestinal flora.^65^ Theaflavins can also exhibit antioxidant activity while improving intestinal flora.^66^ Phloridzin has an anti-aging effect on D-galactose-induced mouse aging through antioxidant and anti-inflammatory activities, prevention of apoptosis, and regulation of intestinal microbiota.^37^

In this study, we have demonstrated that dysbiosis of the gut microbiota is associated with age-related arrhythmia. By restoring downregulated expression of cardiac ion channel proteins, the transplantation of young gut microbiota reduced the susceptibility of arrhythmias in aged mice. This study demonstrates a mechanistic link between the aged gut microbiota and the pathophysiology of arrhythmias. Our results suggested that blocking oxidative stress by modulating the composition of the gut microbiota is a potential anti-arrhythmic approach, particularly in older adults.

### Limitations of the study

More recently, the gut-heart axis hypothesis has been proposed to demonstrate dysfunctional interactions between the microbiota and cardiac arrhythmias. Our current study demonstrated that increased ROS from colon associated with downregulated expression of cardiac ion channel proteins in age-related arrhythmias, providing evidence for the previous recommendations. However, in relation to our findings, there are some problems we need to address for further investigation. There is no clear definition of equivalent human microbiota dysbiosis, which indicates the therapeutic value of FMT based on animal studies. In addition, data on the long-term safety of FMT are lacking, and multiple clinical case reports suggest that FMT treatment can lead to severe infectious disease. Although FMT has potential therapeutic value, it also has some disadvantages and limitations. When using FMT for treatment, it is essential to strictly screen donors, control infection risks, ensure stable transplant outcomes, and fully consider ethical issues and patient safety. FMT requires the use of fecal samples from healthy individuals, so the screening and processing of donors are crucial. If the donor has health issues such as infectious diseases, immune system disorders, or is using antibiotics, it may affect the transplant outcomes and even lead to infections and immune rejection. Although the infection risk of FMT is relatively low, there is still a certain level of risk. If pathogens such as bacteria or viruses are present in the transplanted fecal sample, it may cause infection in the patient. Furthermore, when using fecal samples from non-autologous donors, the infection risk may be even higher. The efficacy of FMT can be influenced by various factors, such as the patient’s intestinal environment, the quality of the transplanted sample, and the method of processing. Therefore, the transplant outcomes may be unstable, and it is difficult to guarantee that every treatment will achieve the desired effect. FMT involves the use of human fecal samples, which raises ethical and moral considerations. For example, how to protect the donor’s privacy and rights, and how to ensure the patient’s informed consent and autonomy. FMT treatment may cause some complications, such as intestinal discomfort, diarrhea, and abdominal pain. Additionally, if the transplanted microbial community does not match the patient’s native microbiota, it may trigger immune reactions or microbiota imbalance. On the other hand, administration of probiotics/prebiotics alleviates systemic oxidative stress and arrhythmogenesis in mice and patients taking aspirin, while antibiotics reduce arrhythmias.^67^^,^^68^ However, antibiotic treatment can destroy the balance of intestinal flora and cannot be used as a long-term treatment strategy. Clearly, further studies are needed to elucidate the mechanisms underlying the adverse changes in gut microbiota during aging. Since estrogen has a protective effect against cardiovascular diseases (including arrhythmias, HF, etc.), female mice are generally not used in the construction of cardiovascular disease models to avoid estrogen interference. In this experiment, the incidence of arrhythmias in aged mice is already relatively low, and there are differences in estrogen levels between aged female mice and young female mice. In order to minimize the impact of factors other than intestinal flora, we prefer to use male mice for the experiment.

## Resource availability

### Lead contact

Further information and requests for resources and reagents should be directed to and will be fulfilled by the lead contact, Zhi-ping Fu (fzy1361351574@126.com).

### Materials availability

This study did not generate new unique reagents.

### Data and code availability

• PCR data reported in this paper will be shared by the lead contact upon reasonable request.
• This study did not generate new original data and code.
• Any additional information required to reanalyze the data reported in this paper is available from the lead contact upon request.

## Acknowledgments

This study was funded by Science Research Project of Hebei Education Department [QN2023059], and Natural Science Foundationin Hebei province of China [H2023209034].

## Author contributions

Z.P.F. designed project, wrote the manuscript, and supervised the whole project; Y.F.Y., R.Y.W., and X.Q.W. helped in data collection and analysis in western blot assay.

## Declaration of interests

The authors declare no competing interests.

## STAR★Methods

### Key resources table

**Table:** 

REAGENT or RESOURCE	SOURCE	IDENTIFIER
**Antibodies**		

anti-connexin 43/GJA1 rabbit antibody	Affinity	Cat# AF0137; RRID: AB_2833319
anti-connexin 40/GJA5 rabbit antibody	Affinity	Cat# DF13633; RRID: AB_2846652
anti-Nav1.5 rabbit antibody	Affinity	Cat# DF13217; RRID: AB_2846236
anti-Kv4.2/KCND2 rabbit antibody	Affinity	Cat# DF7675; RRID: AB_2841147
anti-occludin rabbit polyclonal antibody	HA	Cat# R1510-33; RRID: AB_3073341
anti-CACNA1C rabbit polyclonal antibody	HA	Cat# ER1803-49; RRID: AB_3069245
anti-Serca-2A recombinant rabbit antibody	HA	Cat# ET1703-01; RRID: AB_3070359
anti-ZO-1 rabbit antibody	Affinity	Cat# AF5145; RRID: AB_2837631
anti-IL-10 rabbit antibody	Affinity	Cat# DF6894; RRID: AB_2838853
anti-IL-1β rabbit antibody	Affinity	Cat# AF4006; RRID: AB_2801567
anti-TNF-α rabbit antibody	Affinity	Cat# AF7014; RRID: AB_2835319
anti-IL-6 mouse antibody	Affinity	Cat# DF6087; RRID: AB_2838055
anti-β actin rabbit antibody	Servicebio	Cat# GB11001; RRID: AB_3206256
anti-GAPDH mouse antibody	Servicebio	Cat# GB12002; RRID: AB_3206256
anti-α-SMA rabbit antibody	Affinity	Cat# AF1032; RRID: AB_2835329
anti-p21 rabbit antibody	Affinity	Cat# AF6290; RRID: AB_2827699
anti-p53 rabbit antibody	Affinity	Cat# AF0879; RRID: P04637
anti-bax rabbit antibody	Affinity	Cat# AF0120; RRID: AB_2833304
anti-bcl-2 rabbit antibody	Affinity	Cat# AF6139; RRID: AB_2835021
anti-NRF2 rabbit antibody	Affinity	Cat# AF0639; RRID: AB_2833793
anti-OLA1/GTPBP9 rabbit antibody	Proteintech	Cat# 16371-1-AP; RRID: AB_2157087

**Biological samples**		

CD-1 mice(2/20 months)	Huafukang Biotechnology Co., Ltd.	N/A

**Chemicals, peptides, and recombinant proteins**		

Metronidazole	Bide Pharmaceutical	BD140070
Vancomycin	Bide Pharmaceutical	BD156341
Ampicillin	Bide Pharmaceutical	BD765600
Gentamicin	Bide Pharmaceutical	BD131943
Vitexin	Shyuanye	3681-93-4
DMF	Aladdin	D104459
ML385	Aladdin	M304758
Isoflurane	RWD	R510-22-10
Isoproterenol	Shyuanye	51-30-9
FITC-labeled dextran	Beyotime	ST2930
Vitamin C	Rhawn	26094-91-7
Agarose	Bidepharm	9012-36-6
4% paraformaldehyde	Beyotime	P0099
RIPA	Beyotime	P0013B
PMSF	Beyotime	ST506
Cardiac Troponin I (cTnI) Kit	Beijing Biolead biology technology CO, LTD.	Ctni-1-hs
Creatine kinase-MB (CK-MB) Kit	Wuhan Fine biology technology CO, LTD.	EM0929
MDA	Nanjing Jiancheng Bioengineering Institute,	A003
SOD	Nanjing Jiancheng Bioengineering Institute,	A001
GSH-Px	Nanjing Jiancheng Bioengineering Institute,	A005
Pierce BCA protein assay kit	Thermo Scientific	23225
Lactate dehydrogenase (LDH) Kit	Nanjing Jiancheng Bioengineering Institute,	A020
Fecal DNA extraction Kit	Yeasen	18820ES70

**Deposited data**		

RNA-seq raw and analyzed data	Gene Expression Omnibus	GSE201207 (https://www.ncbi.nlm.nih.gov/geo/query/acc.cgi?acc=GSE201207) GSE245886 (https://www.ncbi.nlm.nih.gov/geo/query/acc.cgi?acc=GSE245886)

**Software and algorithms**		

ImageJ	National Institutes of Health	https://www.statistical-analysis.top/ImageJ/
Image Lab	Bio-Rad Inc.	https://www.bio-rad.com/zh-cn/product/image-lab-software
PHOTOSHOP	Adobe	https://www.adobe.com/products/photosho
GraphPad Prism	Software Inc.	https://www.graphpad.com/
SPSS	SPSS, Inc.	https://www.ibm.com/spss

### Experimental model and study participant details

#### Animal

All procedures involving animals were approved by the Institutional Ethics Committee of North China University of Science and Technology (Approval No. 20230110) and conducted in accordance with the "Animal Research: Reporting of *In Vivo* Experiments" (ARRIVE) guidelines. Male CD-1 mice (8 weeks old), were purchased from the Model Animal Research Center of North China University of Science and Technology, and housed in a specific-pathogen-free (SPF) facility with a 12-h light–dark cycle. Mice were randomly assigned to 4 groups, and housed individually. The number of mice used per experiment was indicated in the figure legends. Investigators performing the data analyses were blinded to the treatment status. For gut microbiota depletion, SPF mice were given free access to water containing a 200 μL cocktail of antibiotics (ABX) [metronidazole (1 mg/mL), vancomycin (1 mg/mL), ampicillin (1 mg/mL), and gentamicin (1 mg/mL) were dissolved in drinking water for 3 days prior to fecal microbiota transplantation (FMT) and for 2 weeks as a gut microbiota depletion model. Animals that received the antibiotic cocktail are referred to as ABX mice, whereas those that received drinking water are referred to as control mice. Body weight was monitored daily. Drinking water and food were replaced daily, and the intake of water and food was documented by measuring the volume and weight before and after replacement. We divided a heart into multiple sections, and only select a small portion of it to perform bacterial counting. The remaining sections can be used for experiments such as paraffin sections, frozen sections, and Western blot (Figure S3).

### Method details

#### Fecal microbiota transplantation

Young (2 months), aged (20 months) male mice were randomly reassigned to experimental cages 1 week before the start of the experiment. The animal experimental protocol is shown in Figure 1A. Mice were randomly divided into 4 groups: i) young-young FMT group (young mice gavaged with autogenous fecal, *n* = 12); ii) young-aged FMT group (the young mice gavaged with fecal from aged mice, *n* = 12); iii) aged-aged FMT group (aged mice gavaged with autogenous fecal, *n* = 12); and iv) aged-young FMT group (the aged mice gavaged with fecal from young mice, *n* = 12). We depleted the host gut microbiota by prior antibiotic cocktail administration to increase engraftment of the donor microbiota. For gut microbiota depletion, SPF mice drank freely water containing a 200 μL cocktail of antibiotics (ABX) [metronidazole (1 mg/mL), vancomycin (1 mg/mL), ampicillin (1 mg/mL), and gentamicin (1 mg/mL)] for 3 days, followed by FMT. For the microbiota suspension preparation, young or aged mice were individually placed in clean cages twice a day and fecal were collected within 20 min. Briefly, fresh feces pellets (200 mg) were collected from young and aged donor mice and placed into autoclaved tubes and homogenized in 5 mL of sterilized phosphate buffered saline (135 mM NaCl, 4.7 mM KCl, 10 mM Na_2_HPO_4_, 2 mM NaH_2_PO_4_, pH7.38), then centrifuged for 3 min at 800 g, and supernatants were transferred to tubes, which was immediately centrifuged (3000 g, 10 min) and then resuspended the precipitate and given to mice by oral gavage. FMT was performed within 20 min from fecal microbiota collection to gavage to minimize the impact of environment on microbiota. Fresh bacterial supernatant was used for each FMT, that was fecal samples from donor mice were prepared on site once collection and then were used for FMT. FMT administration was performed by oral gavage with 200 μL gut microbiota twice a day for a period of 6 weeks. Fecal pellets collections and FMT interventions were all performed at the same time of day across all cages to account for circadian rhythm variability in feeding and microbiota composition. On the final day of the study experiment, the mice were sacrificed under humane methods. The intestine was removed, and its length was measured and photographed. Open the colon and gently press with forceps to remove the fecal contents. The distal portion (approximately 10 mm) of the colon and other sections were selected for the hematoxylin-eosin (H&E) staining, examination of oxidative stress marker, and Western blotting respectively.

#### Fecal parameters measurements

Mice (*n* = 6 for each group) were randomly given food and water and immediately placed in clean cages. They were provided with access to their standard lab chow and tap water *ad libitum*. Then, feces from each mouse were collected, counted, and weighed in 8 o’clock every day. The amount and weight of feces was expressed as the total number and wet weight of feces per mouse. Each heart was divided into three parts, which was used for Western blot and ELISA (stored at −80°C), histological staining (paraffin sections), and DHE staining (frozen sections), respectively.

#### Bacterial recovery

The number of inoculated bacteria and bacterial load (*n* = 6 for FMT treated mice (young-young FMT group, young-aged FMT group, aged-aged FMT group, aged-young FMT group), ABX treated mice (young group, young-ABX group, aged group, aged-ABX group), and vitexin treated mice (aged group, vitamin C group, 60 mg/kg vitexin group, 30 mg/kg vitexin group, 15 mg/kg vitexin group)) in different tissues were determined by viable plate counting method, and the liver, heart, colon, blood, mesenteric lymph node (MLN) and spleen were homogenized. Bacteria present in tissues were quantified by plating serial dilutions on LB agar.

#### DMF, vitexin, and ML385 treatment in aging mice model

Male CD1 mice were treated with a D-gal (150 mg/kg/day for 56 days) to test the anti-aging effects of DMF, vitexin, and ML385. Eight-week-old CD1 mice were divided into four groups: (1) water treated; (2) D-gal (150 mg/kg/day) treated; (3) vitexin (30 mg/kg/day) and D-gal treated; and (4) DMF (30 mg/kg/day) and D-gal treated; (5) ML385 (30 mg/kg/day) and D-gal treated; (6) vitexin, ML385 and D-gal treated. D-gal was dissolved in PBS and administered (150 mg/kg/day) by intraperitoneal injection. DMF was dissolved in PBS with10% DMSO and administered (30 mg/kg/day) by oral gavage. Vitexin was dissolved PBS with 10% DMSO, 40% PEG300, 5% Tween-80, then administered (30 mg/kg/day) by oral gavage. ML385 was dissolved in PBS with 5% DMSO and administered (30 mg/kg/day) by intraperitoneal injection.

#### Estimation of fecal-bacterial loads

To determine fecal bacterial loads, fecal pellets were collected, weighed, and stored at −80°C. DNA was subsequently extracted using the Qiagen DNA Stool Mini kit and quantified using a Nanodrop spectrophotometer. Yields were calculated using DNA per gram of fecal weight and PCR using 16S RNA. We amplified the 16S ribosomal RNA gene of this bacterium by PCR (95°C 10 min followed by 40 cycles at 95°C 15 s, 58°C 30 s, and 72°C 30 s followed by 5 min extension at 72°C using primers (forward and reverse: 5′- actcctacgggaggcagt-3′ and forward and reverse: 5′-attaccgcggctgctggc-3′, where the barcode was an eight-base sequence to each sample). The PCR products were analyzed by 1.2% agarose gel electrophoresis, and the PCR products with the main strip in the range of 180 bp were purified. 16S RNA sequencing data were functionally predicted and calculated using ImageJ.

#### Electrocardiograph

Mice (*n* = 6 for each group, including FMT treated mice, ABX treated mice, H_2_O_2_ treated mice, vitexin and vitamin C treated mice) were anesthetized with mechanical ventilation by inhalation of 1–1.5% isoflurane (RWD, Batch) in 100% oxygen continuously. Surface electrocardiogram (ECG) was monitored by P3 plus (Data Sciences International), 1.5% isoflurane-oxygen mixture was anesthetized, and subcutaneous platinum electrode was placed on lead II. After a 5-min resting ECG detection, 2 mg/kg of isoproterenol was administered via intraperitoneal injection (i.p.), and the subsequent changes in the ECG were continuously monitored. 3-min ECG samples of each animal were analyzed using a specially designed LabChart 9 analysis program, and heart rate, PR interval, R amplitude, and QRS intervals were measured from the 1-min mean curve. Ventricular tachycardia (VT) was defined as at least 4 consecutive premature ventricular beats (PVB).

#### Staining

Fresh heart and proximal colon tissues (*n* = 6 for each group, including FMT treated mice, FMMT treated tissues, ABX treated mice, H2O2 treated mice, vitexin and vitamin C treated mice) were excised and washed in phosphate buffer saline (PBS), and colonic segments at the same location were collected in each mouse and washed in ice-cold PBS buffer. The tissue was fixed with a 4% phosphate buffer paraformaldehyde (pH 7.38), gradually dehydrated, embedded in paraffin, cut into cross sections (5 μm thickness). Then, the tissues were stained with hematoxylin and eosin (H&E), Sirius red, and terminal-deoxynucleotidyl transferase-mediated nick end labeling (TUNEL) staining. In brief, slide was dewaxed and hydrated, then stained with hematoxylin for 3 min and eosin for 30 S respectively. After dehydration, the slide was mounted with xylene and captured image. H&E staining was used to measure the structure of heart and proximal colon. Sirius red staining was to measure the area of fibrosis, and Image-Pro Plus 6.0 software was to quantitative analysis. Collagen volume fraction was calculated by dividing the collagen area by the total area and multiplying by 100%.

#### Biochemical assays

The heart, intestinal and colon tissues was homogenized in pre-cooled phosphate buffered saline (PBS). Then centrifuged and collected the supernatant to test the oxidative stress and antioxidant biomarkers -malondialdehyde (MDA), superoxide dismutase (SOD), and glutathione peroxidase (GSH-Px) levels, and cardiac injury markers-Creatine Kinase-MB (CK-MB) and cardiac Troponin I (cTnI) were determined. Malondialdehyde (MDA), a degradation product of lipid peroxide, can condense with thiobarbituric acid (TBA) to form a red product that has a maximum absorption peak at 532nm. Superoxide anion radical (O₂⁻) is generated through the xanthine and xanthine oxidase reaction system, which oxidizes hydroxylamine to form nitrite. Under the action of the chromogenic reagent, nitrite shows a purplish red color, and its absorbance is measured using a visible light spectrophotometer. When SOD is present in the sample being tested, it has a specific inhibitory effect on superoxide anion radicals, reducing the formation of nitrite. During colorimetry, the absorbance value of the test tube is lower than that of the control tube, and the SOD activity in the tested sample can be calculated. Glutathione peroxidase (GSH-PX) can promote the reaction between hydrogen peroxide (H_2_O_2_) and reduced glutathione (GSH) to produce H_2_O and oxidized glutathione (GSSG). The activity of GSH-PX is expressed by the reaction rate of GSH catalysis.

#### Western blotting

Briefly, fresh heart and colon tissue (*n* = 6 for each group, including FMT treated mice, FMMT treated tissues, ABX treated mice, H_2_O_2_ treated mice, vitexin and vitamin C treated mice) was quickly removed from anesthetized mice and washed with ice-cold PBS, it was then dissolved in ice-cold RIPA buffer containing protease and phosphatase inhibitors (1 mm PMSF and cocktail). Tissue homogenates were incubated on ice for 30 min and then centrifuged at 14,800 rpm for 15 min. The resulting supernatants were collected and quantified with Pierce BCA protein assay kit. To examine the expression different protein, 10–50 μg of protein was loaded on 8%–12% SDS-polyacrylamide gels and transferred to nitrocellulose filter or polyvinylidene fluoride membranes. The membranes were blocked with 5% non-fat milk in TBST at room temperature for 2 h, and then respectively incubated with primary antibodies overnight at 4°C. After washing, the membranes were incubated with the secondary antibodies (Servicebio) for 1 h. The membranes were exposed to ECL buffer after another three washes, and the blots were detected by ChemiDoc XRS gel documentation system (Bio-Rad, Hercules, CA, USA).

#### Eversion of mice small intestine and fecal microbiota metabolites transplantation

Aged (20 months) and young (2 months) mice were randomly divided into 4 groups: i) young-young FMMT group (young mice gavaged with autogenous fecal, *n* = 6); ii) young-aged FMMT group (the young mice gavaged with fecal from aged mice, *n* = 6); iii) aged-aged FMMT group (aged mice gavaged with autogenous fecal, *n* = 6); and iv) aged-young FMMT group (the aged mice gavaged with fecal from young mice, *n* = 6).After deeply anesthetized, the jejunum of the mice intestines (approximately 2 cm) was quickly excised, and the underlying mesenterium was removed. The segment was washed several times with cold normal saline solution and placed immediately into the oxygenated Krebs buffer (NaCl 117 mM, NaHCO_3_ 25 mM, KCl 4.7 mM, CaCl_2_ 2.5 mM, MgCl_2_ 1.2 mM, KH_2_ PO_4_ 1.2 mM, and D-glucose 11 mM). Everted intestine on a gastric perfusion needle (0.5 mm diameter), filled it with 1 mL of Krebs buffer, and dividing it into sacs about 1 cm in length with braided sutures. The sacs were placed in the oxygenated buffer solution (pH 7.38) with added fecal microbial metabolites (1 mL, 1 g fecal to 1 mL PBS). The solution was maintained at 37°C with 95% O_2_ and 5% CO_2_ throughout the experiment. For the microbiota suspension preparation, young or aged mice were individually placed in clean cages twice a day and fecal were collected within 20 min. Briefly, fresh feces pellets (1.2 g) were collected from young and aged donor mice and placed into auto-claved tubes and homogenized in 1.2 mL of sterilized PBS, then centrifuged for 3 min at 5000 g, and 1 mL supernatants were transferred to solution. The sacs were incubated at 37°C for 4 h. The individual sacs were removed, and washed with PBS, and the blots dried. The intestines were selected for H&E staining, oxidative stress marker detection and Western blot detection.

#### Fluorescein isothiocyanate (FITC)-dextran permeability assay

The intestinal permeability was measured *in vivo* using undigested FITC-labeled dextran (4 kDa). Briefly, after overnight water deprivation, FITC-dextran dissolved in PBS (100 mg/mL) was administered via oral gavage (44 mg/100 g) and FITC fluorescence intensity in serum was measured 4 h later. Serum was collected after sacrifice, and blood samples were collected and centrifuged. FITC-dextran was measured using an ultraviolet and visible spectrophotometer (JINGHUA 752, Shanghai, China) at 490 nm. The concentrations of FITC-dextran in the serum were determined by standard curve.

### Quantification and statistical analysis

Continuous variables with a normal distribution were expressed as mean ± standard deviation (SD). Numbers per group in the figure legends refer to the number of animals in each group. Individual data points were only excluded if technical issues were detected during the analysis procedure. Categorical variables were reported as counts and percentages. Differences between percentages were assessed by χ2 test or Fisher’s exact test. The groups with normally distributed parametric data were tested using one-way analysis of variance (ANOVA), followed by Tukey’s (for the same number of samples) or Sidak’s (for different numbers of samples) multiple comparison test (for more than two groups). Non-parametric data were compared by Mann-Whitney’s U test. two-way ANOVA followed by Tukey’s multiple comparison tests was used to determine differences between groups at multiple time points. Variables with *p* < 0.05 at univariable analysis were then included as covariates in multivariable analysis. The Kaplan–Meier method and Log rank (MantelCox) test were used to construct and determine the survival rate between all treatment groups, respectively. All analyses were performed using Prism 9 software (GraphPad Prism Software Inc., San Diego, CA) and SPSS 18 (SPSS, Inc., IL, USA), and all of the statistical details of experiments can be found in the figure legends. Only differences with a *p*-value of less than 0.05 were considered statistically significant. ∗*p* < 0.05, ∗∗*p* < 0.01, ∗∗∗*p* < 0.001, ∗∗∗∗*p* < 0.0001.
