# Supplementary material for: Aged gut microbiota promotes arrhythmia susceptibility via oxidative stress
Source: iScience. 2024 Sep 4;27(10):110888. doi: 10.1016/j.isci.2024.110888 (PMC11460473; doi:10.1016/j.isci.2024.110888)
Supplement: Document S1. Figures S1–S3 [file mmc1.pdf]

iScience, Volume *27*

Supplemental information

Aged gut microbiota promotes arrhythmia susceptibility via oxidative stress

Zhi-ping Fu, Yi-ge Ying, Rui-yao Wang, and Yu-qing Wang

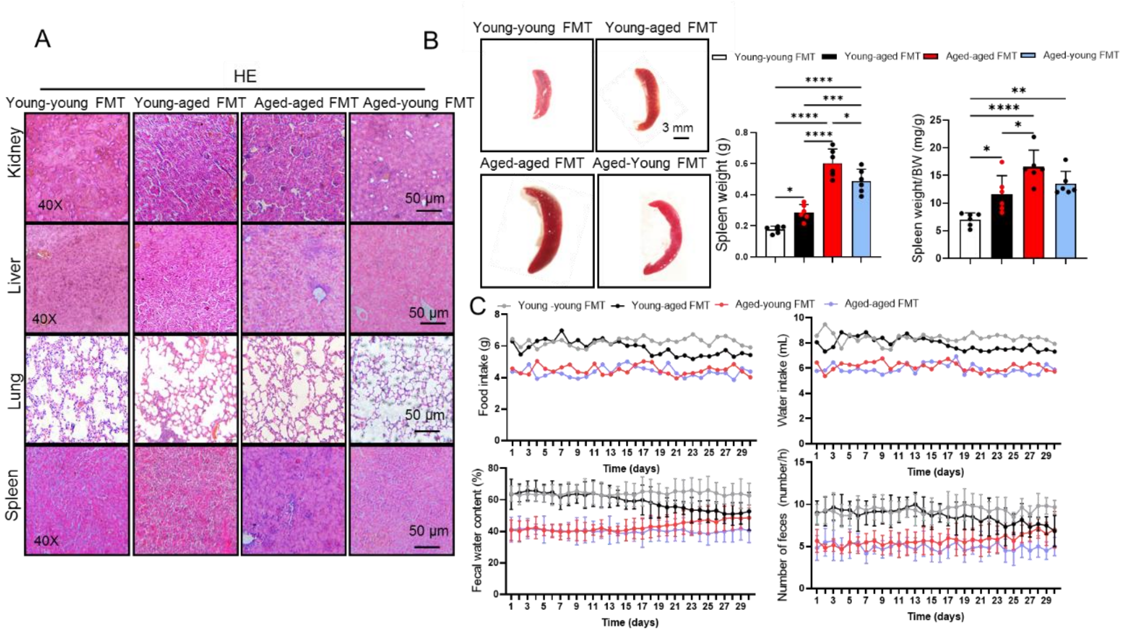

Figure S1. The effect of FMT on aging process, Related to figure 1.

(A) Representative photographs of H&E staining of kidney, liver, lung, spleen tissues of young-young FMT, young-aged FMT, aged-aged FMT, aged-young FMT mice (n= 6/ per group).

(B) The overall spleen size. Scale bar is 3 mm. Quantitative analysis of spleen weight, spleen/body weight in the different groups (n= 6/ per group). Data analyzed by one-way ANOVA with Tukey's post-hoc test.

(C) Broken line represent average value of food and water intake amount of each group. Fecal water content and number of feces from mice in the young-young FMT, young-aged FMT, aged-aged FMT, aged-young FMT groups.

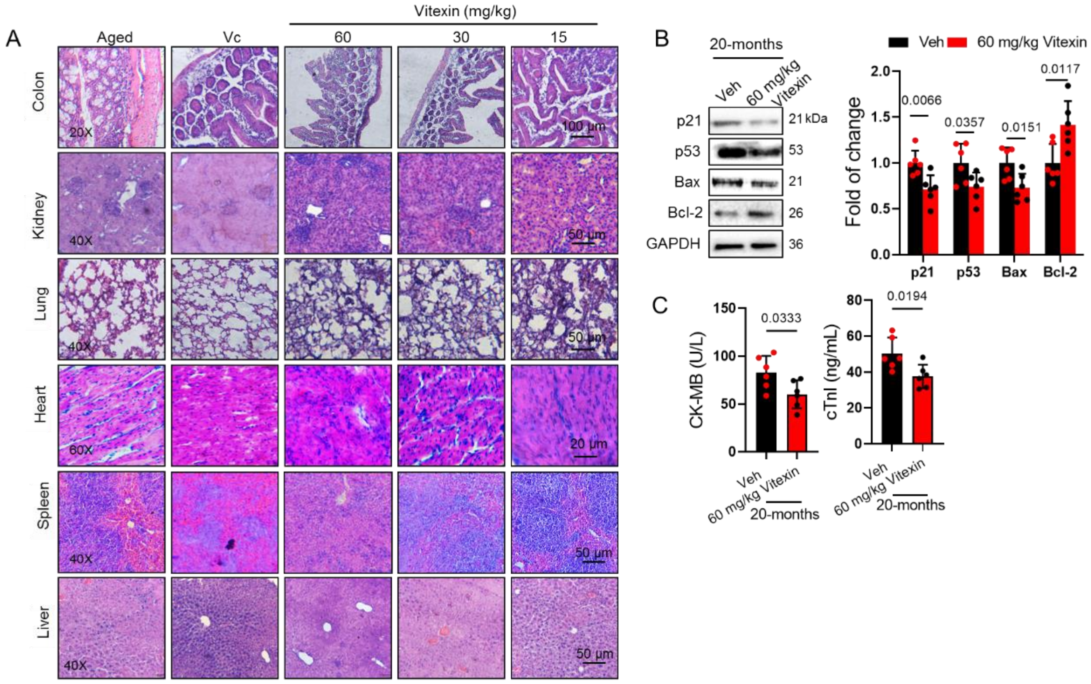

Figure S2. The effect of vitexin on aging process, especially for heart, Related to figure 9.

(A) Representative photographs of H&E staining of colon, heart, kidney, liver, lung, and spleen tissues from mice treated with vehicle, vitamin C, and different doses (60, 30, 15 mg/ kg) of vitexin.

(B) Representative Western Blot and quantification of p21, p51, bax, bcl-2, GAPDH in ventricular aged, and vitamin C treated aged mice (n= 6/ per group). GAPDH was used for internal normalization. Data analyzed by unpaired two tailed Student’s t test.

(C) Cardiac Creatine Kinase-MB (CK-MB), Cardiac Troponin I (cTnI) levels were quantified using commercial assay kits (n= 6/ per group). Data analyzed by unpaired two tailed Student’s t test.

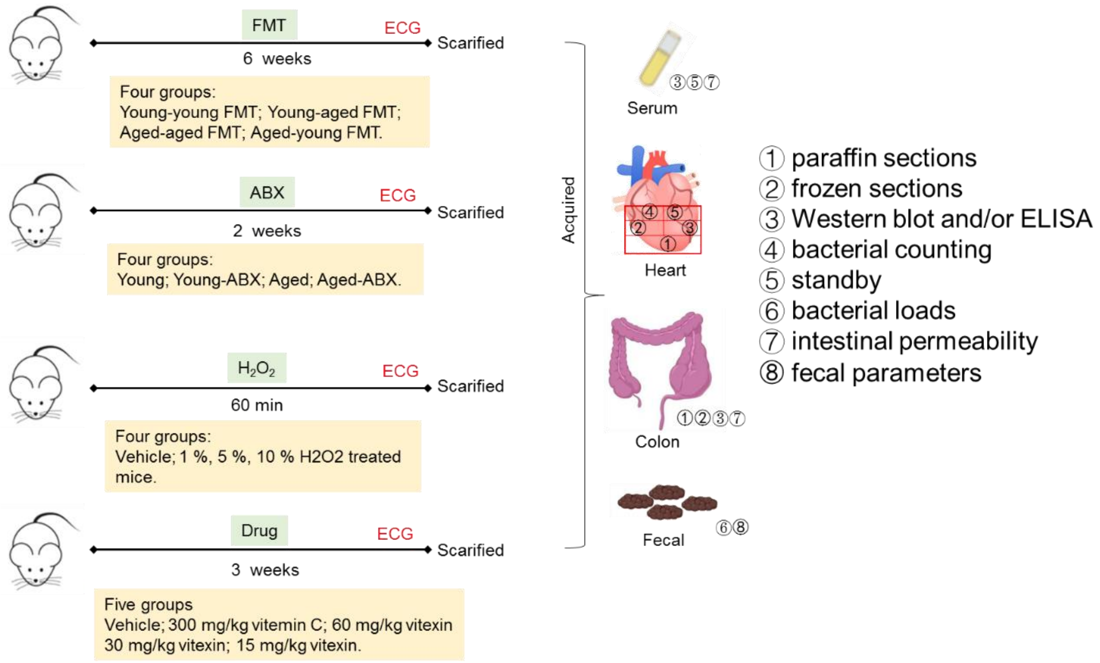

Figure S3. A heart was divided into multiple sections, and only select a small portion of it to perform bacterial counting. The remaining sections can be used for experiments such as paraffin sections, frozen sections, and Western blot, Related to figure 9.
